# Synergistic Enhancement Effect of Maxwell Polarization and Preferential Exposure of Zn (101) Plane toward Superhydrophobic Separator for Ah‐Level Zinc Metal Pouch Batteries

**DOI:** 10.1002/advs.202506035

**Published:** 2025-07-30

**Authors:** Xinyao Yuan, Di Zhang, Hongfei Lu, Yuhang Song, Zhiyi Du, Minjie Song, Nawei Lyu, Yang Jin

**Affiliations:** ^1^ Research Center of Grid Energy Storage and Battery Application School of Electrical and Information Engineering Zhengzhou University Zhengzhou Henan 450001 China

**Keywords:** maxwell polarization, separator, uniform electric field, zinc metal batteries, Zn (101) oriented deposition

## Abstract

Interfacial instability in aqueous zinc metal batteries (AZMBs) leads to uncontrolled dendrite growth and water‐induced parasitic reactions, limiting their practical applications. In this work, a novel superhydrophobic HfO_2_‐coated functionalized glass fiber separator is proposed. Characterized by its high dielectric constant, HfO_2_ can autonomously generate an oriented electric field under external electric field stimulation, ensuring uniform distribution of the electric field and Zn^2+^ flux at the interface. This strategy not only enhances Zn^2+^ transport kinetics but also effectively suppresses the migration of SO_4_^2−^ via electrostatic repulsion. Furthermore, the HfO_2_@GF separator promotes the deposition of Zn^2+^ along the Zn (100) and Zn (002) crystal planes, facilitating the preferential exposure of the unique Zn (101) crystal plane. Additionally, its superhydrophobic property effectively inhibits interfacial side reactions. Therefore, the symmetrical cell exhibits an ultra‐long cycling life of 4660 h at 5 mA cm^−2^ and 1 mAh cm^−2^, and maintains stable operation up to 5050 h at 10 mA cm^−2^. The Zn||I_2_ full battery maintains 87.75% of its initial capacity after 8000 cycles at 10C. Furthermore, the stacked Zn||I_2_ pouch battery provides an Ah‐level capacity (1.64 Ah). This work provides a new insight into the construction of highly stable interfacial chemistry.

## Graphical abstract

A superhydrophobic HfO2‐coated glass fiber separator is proposed for long‐life zinc‐ion batteries. Under the stimulus of the external electric field, the separator autonomously generates a directional electric field, ensuring uniform distribution of the electric field and Zn^2+^ flux. Additionally, the separator promotes the preferential exposure of Zn (101) crystal plane, and its superhydrophobic properties suppress side reactions, improving interface stability.

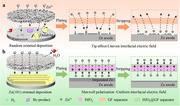

## 1 Introduction

Aqueous zinc metal batteries (AZMBs) are considered as a promising alternative to lithium‐based energy storage devices due to their high theoretical capacity (820 mAh g^−1^ and 5855 mAh cm^−3^), abundant resources, intrinsic safety, and cost‐effectiveness.^[^ ^1^, ^2^, ^3^ ^]^ Compared with other metal‐ion batteries (Na, K, Mg, and Al), Zn‐based systems have shown rapid development due to their excellent compatibility with aqueous electrolytes.^[^ ^4^ ^]^ However, the application of Zn metal anodes in aqueous electrolytes faces some unavoidable inherent challenges. On the one hand, the surface of commercial zinc foil, marked by residual manufacturing defects (tiny tips), induces uneven electric field distribution. This results in preferential nucleation of Zn^2+^ at high‐energy sites. Over extended cycles, this can result in the formation of localized disordered Zn^2+^ deposits, commonly known as zinc dendrites.^[^ ^5^, ^6^ ^]^ Such behavior further disrupts the uniformity of interfacial electric fields and ion fluxes, with the issue worsening under higher current densities. On the other hand, the high activity of water decomposition causes local changes in the electrolyte pH, leading to continuous hydrogen evolution reactions (HER) and self‐corrosion, which result in battery swelling and Zn anode passivation.^[^ ^7^, ^8^ ^]^ These problems will lead to shortened cycle life, reduced reversibility, capacity degradation, and battery failure.

To address these issues, researchers have proposed various strategies, including electrolyte regulation, anode interface modification, and anode structure design. Electrolyte regulation includes the design of high‐concentration electrolytes (3 M Zn(ClO_4_)_2_+0.3 M Zn(OAc)_2_,^[^ ^9^ ^]^ 5 M Zn(ClO_4_)_2_^[^ ^10^ ^]^), the use of different electrolyte additives (yttrium 2,4,5‐trifluorophenylacetate,^[^ ^11^ ^]^ sodium alginate,^[^ ^12^ ^]^ acetamide,^[^ ^13^ ^]^ H_2_SO_4_^[^ ^14^ ^]^), the construction of gradient electrolytes,^[^ ^15^ ^]^ and gel electrolytes.^[^ ^16^, ^17^ ^]^ Anode interface modification mainly involves the in situ or ex situ establishment of various protective coatings on the Zn metal surface, including oxides (HfO_2_,^[^ ^18^, ^19^ ^]^ ZrO_2_^[^ ^20^ ^]^), polymers (cPAN^[^ ^21^ ^]^), carbon materials (Gr^[^ ^22^ ^]^), metal‐organic frameworks (Co‐ZIF‐8^[^ ^23^ ^]^), and metals or alloys (Ag,^[^ ^24^ ^]^ AgZn_3_^[^ ^25^ ^]^). The anode structure design includes alloyed Zn anode,^[^ ^26^ ^]^ (002) crystal plane preferential orientation deposition,^[^ ^27^ ^]^ constructing the 3D structure anode,^[^ ^28^ ^]^ and cold isostatic pressing to create surface microcracks.^[^ ^29^ ^]^ However, few studies have focused on modification of the separator to improve the electrochemical performance of the Zn anode. As an indispensable component of the battery, the separator plays a dual role in physically isolating the electrodes and facilitating ion transport.^[^ ^30^ ^]^ Currently, glass fiber (GF) with good electrolyte wettability is widely used as the separator in AZMBs. However, GF separators typically exhibit large, non‐uniform pore sizes and poor mechanical strength. This results in uneven ionic transport, enabling Zn^2+^ to preferentially accumulate at specific sites and form zinc dendrites over extended plating/stripping cycles. These dendrites may eventually puncture the separator, leading to battery failure.^[^ ^31^, ^32^ ^]^ In addition, excessive hydrophilicity can lead to structural collapse of the separator and promote hydrotropic side reactions.

The development of functionalized separators plays an important role in regulating the ion behavior at the electrode/electrolyte interface. Studies have shown that the deposition behavior of Zn^2+^ largely depends on the distribution of the electric field at the interface.^[^ ^33^ ^]^ The uniform distribution of the electric field facilitates homogeneous ion flux, which enhances uniform nucleation and improves zinc plating/stripping performance. Based on these findings, extensive research has been conducted on separators, including Cu‐coated separators,^[^ ^31^ ^]^ DIE separators,^[^ ^34^ ^]^ AgNWs/BC,^[^ ^35^ ^]^ ZIF‐8‐GF,^[^ ^36^ ^]^ Zr‐CNF,^[^ ^37^ ^]^ PAN separator,^[^ ^38^ ^]^ and Janus separator.^[^ ^33^ ^]^ These separator modification strategies have demonstrated potential in achieving uniform zinc deposition, though further optimization is required. In addition, the deposition pattern of Zn^2+^ is affected by the orientation of the deposited crystals.^[^ ^39^ ^]^ Currently, the construction of Zn (002) crystalline surfaces has emerged as the preferred approach for achieving planar zinc deposition layers, attributed to its lower surface energy, superior corrosion resistance, and dendrite‐inhibiting properties.^[^ ^40^, ^41^ ^]^ However, recent studies have revealed that the Zn (002) crystal plane generally demonstrates relatively weak bond interactions with the deposited atoms. As a result, zinc deposited on Zn (002) lattice textures tend to face challenges in sustaining growth along the original crystallographic direction during prolonged cycling. This results in enhanced lattice distortion during subsequent deposition cycles.^[^ ^42^, ^43^ ^]^ The Zn (002) crystal plane fails to sustain continuous planar epitaxy under repetitive electroplating/stripping cycles. In contrast, the Zn (101) crystal plane, characterized by higher surface energy and reduced lattice distortion, exhibits excellent dendrite‐free zinc deposition behavior, demonstrating notable superiority.^[^ ^43^, ^44^ ^]^ Therefore, there is an urgent demand for the development of a separator modification strategy capable of simultaneously suppressing side reactions and ensuring uniform zinc deposition throughout the cycling process.

Herein, we prepare a superhydrophobic HfO_2_‐coated glass fiber separator (HfO_2_@GF) using a simple spin‐coating process to create a highly stable, dendrite‐free Zn metal anode. During the electrochemical cycling process, the inorganic material HfO_2_, with a high dielectric constant, exhibits significant space charge separation under an external electric field, resulting in an ordered alignment of electric dipoles. These dipoles can generate a locally enhanced and uniformly distributed built‐in directional electric field at the electrode/electrolyte interface through the Maxwell polarization effect. The resulting polarization electric field not only homogenizes the interfacial electric field distribution but also hinders the movement of harmful anions via electrostatic repulsion, thereby suppressing the formation of by‐products related to SO_4_^2−^. Experimental results and theoretical calculations demonstrate that, compared to the Zn (100) and Zn (002) crystal planes, the HfO_2_@GF separator induces Zn^2+^ to exhibit the lowest deposition rate on the (101) crystal plane. This promotes the preferential exposure of the Zn (101) crystal plane, facilitating the formation of a highly ordered and dense zinc deposition layer. In addition, unlike conventional hydrophilic separators, the HfO_2_@GF separator exhibits hydrophobic properties, which effectively suppress water‐induced parasitic reactions. More importantly, the HfO_2_@GF separator allows the passage of small amounts of bound water molecules, ensuring adequate wetting of the interface. Moreover, the inorganic ceramic material HfO_2_ can serve as a robust physical barrier, giving the separator good mechanical properties to resist the vertical growth of dendrites. Benefit from the synergistic regulation of the effects mentioned above, the zinc symmetric battery assembled with the HfO_2_@GF separator demonstrates an ultra‐long zinc plating/stripping lifespan of 4660 h (over 6 months) at a current density of 5 mA cm^−2^ and a capacity of 1 mAh cm^−2^. Surprisingly, even at a high current density of 10 mA cm^−2^ and 1 mAh cm^−2^, the battery can operate stably for 5050 h, which is significantly better than the battery based on the GF separator (199 h). In addition, when the HfO_2_@GF separator is coupled with a thin zinc metal anode (20 µm) and a high‐loading I_2_ cathode, the assembled Zn||I_2_ full battery shows excellent cycling stability. After 8000 cycles at 10C, it still maintains a high capacity retention of 87.75%. More importantly, the assembled stacked Zn||I_2_ pouch battery can deliver an Ah‐level capacity (1.64 Ah) and good cycling stability, with a capacity retention rate of 83.77% after 90 cycles. This work combines dielectric engineering, crystal surface modulation, and interfacial hydrophobicity modulation to provide a new paradigm for realizing high‐performance AZMBs.

## 2 Results and Discussion

### 2.1 Physical and Chemical Characterization of HfO_2_@GF Separator

**Figure 1:**
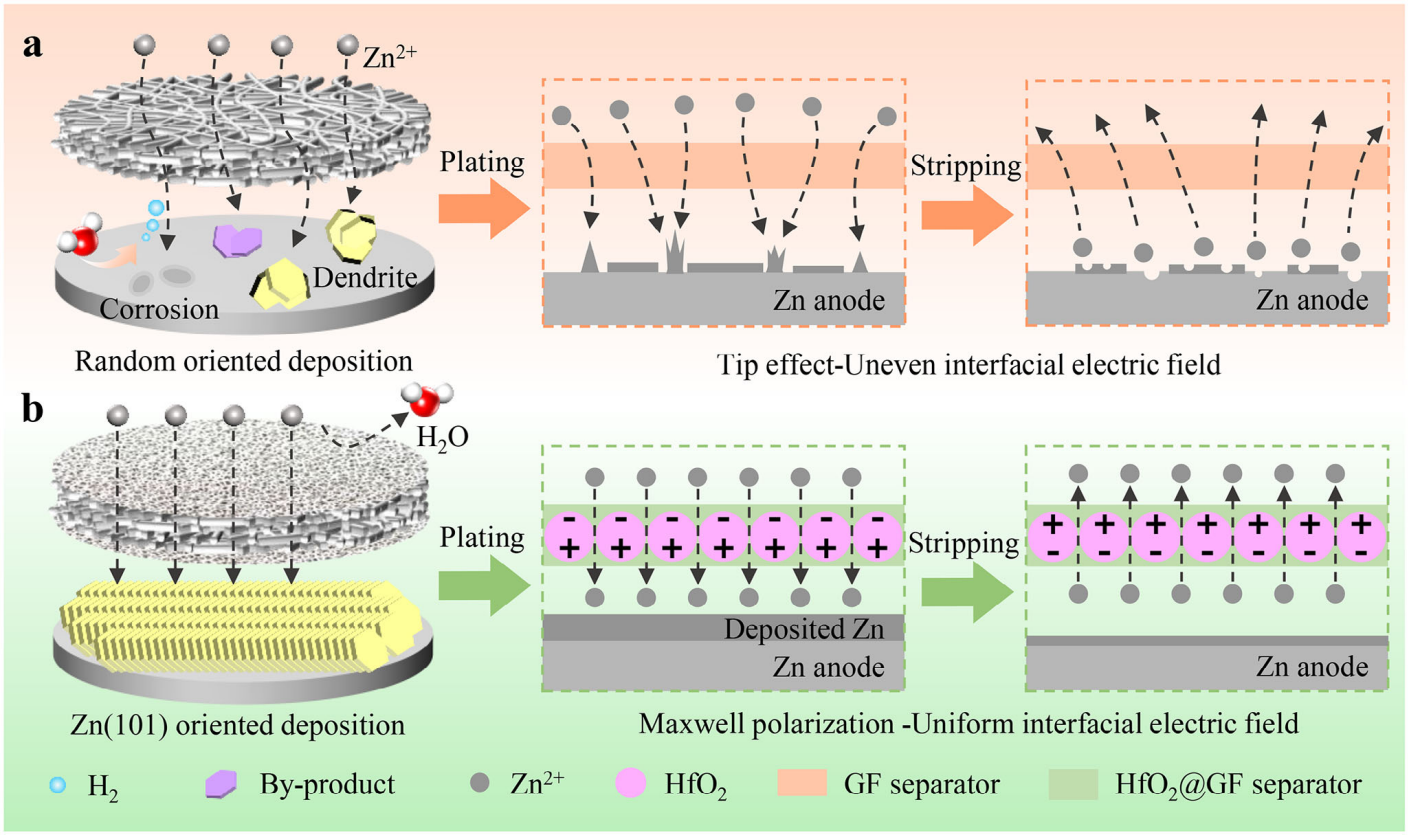
Schematic diagram of the regulation mechanism of different separators on the electrodeposition behavior of zinc anode. a) Deposition behavior of zinc anode with GF separator and b) HfO_2_@GF separator.

In a conventional hydrophilic GF separator, due to the uneven and relatively large pore structure, the solid‐liquid interface is typically accompanied by an uneven electric field distribution. This results in the accumulation of Zn^2+^ in regions with higher electric field strengths, which subsequently generates numerous irregular nucleation sites (**Figure** **1**a). During the subsequent electroplating process, Zn^2+^ tends to deposit preferentially at these high‐energy sites due to their higher surface energy. Over time, this cumulative process leads to the formation of zinc dendrites, whose excessive growth can eventually penetrate the separator, resulting in rapid battery failure. Furthermore, the excessive hydrophilicity of GF separator promotes the accumulation of highly reactive water molecules at the negative electrode interface, thereby triggering HER, corrosion, and unwanted by‐products. In contrast, in the HfO_2_@GF separator system with uniform pores (Figure 1b), the high dielectric constant material HfO_2_ can respond to the external electric field. Under the influence of the external electric field, the positive charge centers of the HfO_2_ particles shift toward the direction of the electric field, whereas the negative charge centers migrate in the opposite direction. This process generates a series of ordered electric dipoles. The electric dipoles can induce a uniformly distributed polarized electric field at the anode interface. During the plating process, the direction of the polarized electric field points to the Zn anode, and on the contrary, during the stripping process, the direction of the polarized electric field points to the cathode.^[^ ^45^ ^]^ The directional electric field induced by HfO_2_, a high dielectric constant material, effectively homogenizes the interfacial electric field via Maxwell polarization. In addition, the introduction of HfO_2_ facilitates the exposure of distinct Zn (101) crystalline surfaces, which promotes uniform and orderly Zn electrodeposition and effectively mitigates the “tip effect”. Notably, the superhydrophobic property of the HfO_2_@GF separator significantly reduces the concentration of highly active water molecules near the anode. This minimizes contact between the Zn electrode and water molecules, thereby effectively reducing water‐induced side reactions.

**Figure 2:**
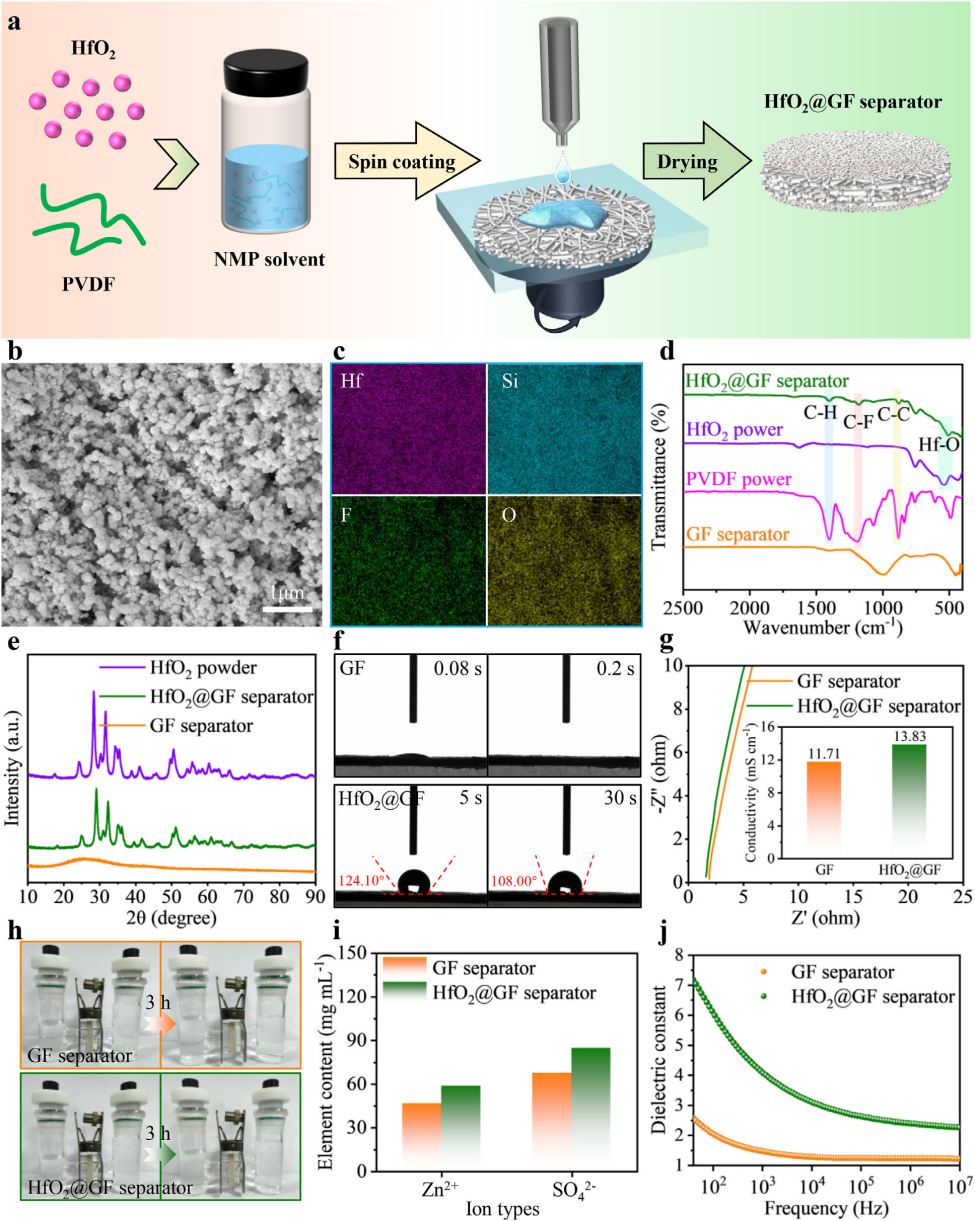
Preparation and structural characterization of HfO_2_@GF separator. a) The simple preparation process of the HfO_2_@GF separator. b) The SEM morphology of the surface and c) the EDS mapping of the corresponding elements of the HfO_2_@GF separator. d) The FTIR spectra and e) the XRD patterns of the GF separator and the HfO_2_ separator. f) Dynamic contact angle test of 2 m ZnSO_4_ electrolyte on the surface of GF separator and HfO_2_@GF separator. g) Nyquist plots of SS||SS batteries with different separators. (Inset: Ionic conductivity of different separators in 2 m ZnSO_4_ electrolyte. h) Ion permeation test of different separators. (Left side: 8 mL of 2 m ZnSO_4_ electrolyte. Right side: 12 mL of deionized water.) i) Ion concentration in the right chamber of the H‐type electrolyzer. j) The dielectric constants of the GF separator and the HfO_2_@GF separator.

The HfO_2_@GF separators are prepared by spin‐coating HfO_2_@PVDF slurry onto commercial GF separators, **Figure** **2**a illustrates the simple preparation process of the HfO_2_@GF separator. To determine the optimal doping ratio of HfO_2_ and PVDF, we prepare HfO_2_@GF separators with HfO_2_ and PVDF mass ratios of 9:1, 7:3, and 5:5, respectively. The cycling performance of symmetric batteries based on HfO_2_@GF separators with different doping ratios is investigated (Figure S1, Supporting Information). Among the tested configurations, the HfO_2_@GF separator with a 9:1 mass ratio exhibits the most significant improvement in cycle life, leading us to select this optimized configuration for further studies. Furthermore, to clarify the synergistic mechanism between HfO_2_ and PVDF, we prepare GF separators containing only PVDF (denoted as pure PVDF@GF separator) and only HfO_2_ (denoted as pure HfO_2_@GF separator). The microscopic surface morphology of the separators is characterized by scanning electron microscopy (SEM). As shown in Figure S2 (Supporting Information), the pristine GF separator consists of randomly oriented amorphous silica fibers with varying sizes and lengths, forming a network structure characterized by large pores (µm level) and irregularities. This leads to the excessive permeation of a large number of free water molecules. The pure PVDF@GF separator exhibits an extremely dense and non‐porous microstructure, which is unfavorable for ion transport (Figure S3a,b, Supporting Information). Additionally, due to the lack of the fixing effect of the PVDF binder, in the pure HfO_2_@GF separator, HfO_2_ particles are loosely accumulated on the surface of the GF separator, which affects the uniform distribution of ion flux (Figure S3c,d, Supporting Information). In contrast, benefiting from the synergistic effect between the PVDF binder and HfO_2_ nanoparticles, the prepared HfO_2_@GF separator exhibits a uniformly dense nanoscale pore structure (Figure 2b). This uniform and abundant porous structure not only facilitates the homogenization of Zn^2+^ flux, enabling uniform zinc plating/stripping but also creates favorable conditions for the full wetting of the electrolyte, providing more transport channels for Zn^2+^.^[^ ^3^ ^]^ In addition, as shown in Figure S4 (Supporting Information), the cycle life of Zn||Zn batteries with pure PVDF@GF separator and pure HfO_2_@GF separator is significantly lower than that of HfO_2_@GF separator. This further validates the significant advantage of the synergistic modulation of PVDF and HfO_2_.

Further observation of the elemental distribution as shown in Figure 2c reveals that the Hf, O, F, and Si elements are uniformly distributed, indicating that HfO_2_ and PVDF are uniformly dispersed on the surface of the GF separator. In addition, the cross‐sectional SEM images of the separator and the corresponding energy dispersive spectroscopy (EDS) images further confirm that HfO_2_ and PVDF are uniformly distributed on both sides of the GF separator (Figure S5, Supporting Information). To further investigate the structural composition of the separator, a Fourier Transform Infrared (FTIR) spectroscopic test is performed. As shown in Figure 2d, the absorption peaks at 423.34 and 999.93 cm^−1^ correspond to the symmetric stretching vibration of Si−O and the asymmetric stretching vibration of Si−O−Si, respectively.^[^ ^32^ ^]^ The characteristic peaks of HfO_2_@GF separator at 1401.54, 1190.70, and 882.69 cm^−1^ match well with the functional group vibration peaks (C─H, C─F, C─C) of PVDF powder. In addition, a prominent absorption peak observed at 539.92 cm^−1^ corresponds to the stretching vibration of Hf‐O. This confirms the successful preparation of the HfO_2_@GF separator. Further, the crystalline phase of the HfO_2_@GF separator is characterized by X‐ray diffraction (XRD) mapping (Figure 2e). The original GF separator exhibits a broad diffraction peak near 25.4°, corresponding to amorphous silica. The HfO_2_@GF separator exhibits obvious diffraction peaks at 29.1° and 32.3°, which correspond to the diffraction peaks of HfO_2_, indicating that HfO_2_ has been successfully introduced into the GF separator. In addition, due to the amorphous nature of PVDF, no corresponding characteristic peaks are observed.

Unlike conventional hydrophilic GF separators, the HfO_2_@GF separator exhibits remarkable hydrophobicity. As shown in Figure 2f, when a drop of 2 m ZnSO_4_ electrolyte is added to the separator, the electrolyte is rapidly absorbed by the GF separator. This indicates that the GF separator exhibits superhydrophilicity, which is mainly attributed to the large pore size of the GF separator. As shown in Figure S6 (Supporting Information), the pure PVDF@GF separator exhibits a high contact angle of 140.99°, demonstrating extreme hydrophobicity, which is unfavorable for electrolyte penetration. Moreover, its highly dense pore structure further exacerbates this disadvantage, significantly hindering ion transport. In contrast, the pure HfO_2_@GF separator shows a contact angle similar to that of the GF separator (0°), indicating that HfO_2_ itself lacks hydrophobicity. This could lead to the accumulation of a large number of free water molecules near the interface, potentially triggering side reactions. In contrast, through the synergistic regulation of PVDF and HfO_2_, the prepared HfO_2_@GF separator exhibits a contact angle of 124.10° after 5 s, indicating that the HfO_2_@GF separator is hydrophobic. Furthermore, as time progresses, the contact angle gradually decreases to 108.00° at 30 s (Figure 2f). This fully verifies that the HfO_2_@GF separator can block the passage of a large number of free water molecules in the electrolyte while allowing the passage of partially bound water molecules to transport the electrolyte.^[^ ^46^ ^]^ This not only significantly inhibits the deleterious interfacial side reactions induced by highly reactive free water molecules. More importantly, the presence of partially bound water molecules ensures sufficient wetting of the separator for Zn^2+^ transport. Furthermore, it is investigated that this contact angle (108.00°) exhibits the maximum value among the currently reported separators for aqueous zinc‐based batteries (Figure S7, Supporting Information).

The ionic conductivity of stainless steel (SS)||SS batteries with GF and HfO_2_@GF separators are measured by electrochemical impedance spectroscopy (EIS). As shown in Figure 2g and Table S1 (Supporting Information). Interestingly, the HfO_2_@GF separator has an improved ionic conductivity compared to the GF separator, indicating that the introduction of the hydrophobic coating does not have a significant effect on the ionic conductivity. This is attributed to the uniform and abundant porous structure of the HfO_2_@GF separator and the bound water permeation. To further verify the water barrier ability of the HfO_2_@GF separator, we conduct ion permeability tests in H‐type electrolytic cells with different separators (Figure 2h). It is worth noting that after standing for 3 h, the left and right chambers of the electrolytic cell with the GF separator exhibit nearly identical liquid levels. This indicates that the GF separator cannot block the passage of water molecules. In contrast, the electrolytic cell with the HfO_2_@GF separator still shows a clearly visible difference in liquid level height after the same time of resting. This visually demonstrates the excellent ability of the HfO_2_@GF separator to isolate water. In addition, further observations show that, compared to the initial time, the liquid level difference in the HfO_2_@GF separator significantly decreases. This indicates that the HfO_2_@GF separator is not completely hydrophobic and still possesses some permeability. Furthermore, the molecular‐level control of water transport by the separators is further analyzed using Raman spectroscopy. The O─H stretching vibrations in the 2800 cm^−1^ to 3800 cm^−1^ region can be divided into three characteristic peaks, corresponding to strong hydrogen bond, medium hydrogen bond, and weak hydrogen bond, respectively.^[^ ^47^ ^]^ As shown in Figure S8 (Supporting Information), compared to the GF separator, the peak area corresponding to a strong hydrogen bond in the right chamber solution with the HfO_2_@GF separator decreases (from 49.91% to 42.07%). This suggests that a large number of water molecules in the solution participate in the formation of the [Zn(H_2_O)_6_]^2+^ complex.^[^ ^44^ ^]^ This is attributed to the fact that the HfO_2_@GF separator can block the penetration of free water while allowing the passage of bound water. This provides molecular‐level evidence for the effect of the HfO_2_@GF separator on water transport.

In addition, we use permeation tests to study the impact of the HfO_2_@GF separator on ion transport. In the H‐type electrolysis cell, equal volumes of 2 M ZnSO_4_ solution and deionized water are separated by the GF separator and the HfO_2_@GF separator (Figure S9, Supporting Information). After standing for 30 min, we measure the ion concentration in the solution on the right side of the electrolysis cell using inductively coupled plasma optical emission spectroscopy (ICP‐OES) to reveal the effect of different separators on ion diffusion. The results show that the electrolysis cell with the HfO_2_@GF separator in the right chamber exhibits higher concentrations of Zn^2+^ and SO_4_^2−^ than the cell with the GF separator (Figure 2i), indicating that the HfO_2_@GF separator has superior intrinsic ion transport capabilities. Furthermore, a constant current is applied to the H‐type electrolysis cell. After 30 min of electrolysis, we collect the solution in the right chamber and determine the ion concentration by ICP‐OES. As shown in Figure S10 (Supporting Information), the Zn^2+^ concentration in the right chamber with the HfO_2_@GF separator is significantly higher than that with the GF separator, while the SO_4_^2−^ concentration is lower than that with the GF separator. This demonstrates that under the influence of the Maxwell electric field, the HfO_2_@GF separator exhibits efficient transmission of Zn^2+^ and inhibits the migration of SO_4_^2−^.

The mechanical properties of the separators are critical to inhibit zinc dendrite penetration.^[^ ^48^, ^49^ ^]^ The mechanical properties of the separators are evaluated using the universal tensile tester. As shown in Figure S11 (Supporting Information), the tensile strength (0.46 MPa) and elongation at break (0.62%) of HfO_2_@GF separator are significantly better than those of GF separator (3.65 MPa, 2.67%). Additionally, SEM analysis of the separator after cycling further confirms that the HfO_2_@GF separator shows no significant changes after long‐term charging and discharging, maintaining its structural integrity. In contrast, due to excessive hydrophilicity and lower mechanical strength, the original GF separator has completely adhered to the Zn anode, with severe fiber fracture on the surface and obvious structural collapse (Figure S12, Supporting Information). This indicates that its high mechanical strength effectively resists the growth stress of zinc dendrites, thereby ensuring the long‐term cycling stability of the battery.

The dielectric constant is a key physical parameter that characterizes the ability of a material to polarize under an applied electric field.^[^ ^50^ ^]^ The dielectric constant of the separator is evaluated by broadband dielectric spectroscopy as shown in Figure 2j. Compared to the GF separator, the dielectric constant of the HfO_2_@GF separator increases significantly to 7.17 (40 Hz). The phenomenon where the dielectric constant decreases with increasing frequency corresponds to the lag of the polarization process, which typically exhibits a relaxation process. This enhanced dielectric property manifests as HfO_2_ forming oriented dipoles through Maxwell polarization under the external electric field, thereby generating a depolarizing internal oriented electric field opposite to the applied electric field.^[^ ^51^ ^]^ For the same applied electric field strength (***E***), the increase in the relative dielectric constant (ε_*r*_) leads to the enhancement of the polarization strength (***P***).^[^ ^50^, ^52^, ^53^, ^54^ ^]^ This indicates that high dielectric material has stronger polarizability and larger polarization field strength (***E_P_***). Therefore, compared to the GF separator, the HfO_2_@GF separator with a high dielectric constant can induce a stronger internal oriented electric field.^[^ ^55^ ^]^ This helps to accelerate the transmission of Zn^2+^ and effectively suppress the aggregation of SO_4_^2−^ on the electrode surface through electrostatic repulsion.

### 2.2 Effect of HfO_2_@GF Separator on Interfacial Behavior

**Figure 3:**
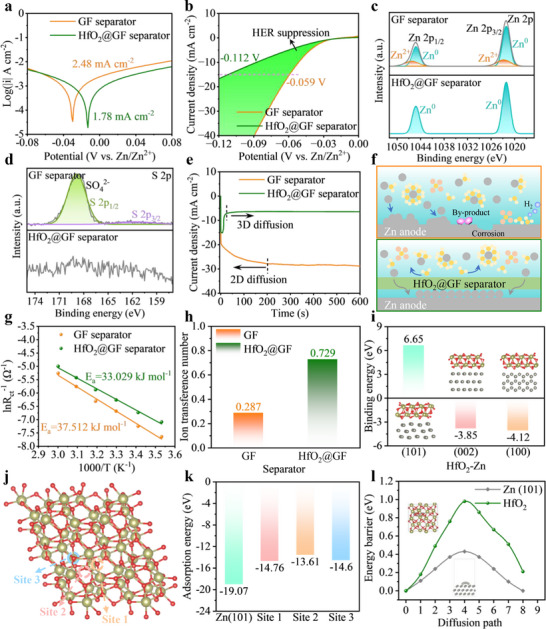
Effect of HfO_2_@GF separator on interfacial behavior. a) Tafel curves for GF separator and HfO_2_@GF separator. b) LSV tests for GF separator and HfO_2_@GF separator in 2 m Na_2_SO_4_ solution. XPS patterns of c) Zn 2p and d) S 2p of zinc anodes with GF separator and HfO_2_@GF separator after 50 cycles. e) CA test of Zn||Zn batteries with different separators at −150 mV fixed potential. f) Schematic illustration of the diffusion of Zn^2+^ near the zinc anode with GF separator and HfO_2_@GF separator. g) Activation energies of different separators obtained based on the Arrhenius equation. h) The number of Zn^2+^ ions transferred of Zn||Zn batteries with different separators. i) The interface formation energy of HfO_2_ with different zinc crystal faces. j) Possible adsorption sites for Zn^2+^ on HfO_2_ crystals. k) Adsorption energy of Zn^2+^ on Zn (101) crystal faces and HfO_2_ crystals. l) Zn^2+^ diffusion properties barriers on the Zn (101) surface and in HfO_2_ crystals (inset shows the corresponding diffusion paths).

To verify the protective effect of the HfO_2_@GF separator on the electrode/electrolyte interface, Tafel curve is tested to assess the corrosion resistance of the zinc anode (**Figure** **3**a). Compared to the pristine GF separator, the Zn anode with HfO_2_@GF separator shows higher corrosion potential (‐10.17 mV) and lower corrosion current density (1.78 mA cm^−2^), demonstrating a substantial reduction in its corrosion tendency. To quantify the inhibitory effect of HfO_2_@GF separator on the corrosion behavior of Zn anode, we quantitatively calculate the corrosion rate of the Zn anode by monitoring the change in the potential of the Zn||Zn@Ti battery over time. As shown in Figure S13 (Supporting Information), the potentials of the batteries with GF and HfO_2_@GF separator increase significantly at 74 and 160 h, respectively, which means that the Zn deposited on the Zn@Ti electrode has been completely consumed due to the corrosion reaction. The calculated zinc anode corrosion rate for the HfO_2_@GF separator (0.0081 mg h^−1^) is significantly lower than that of the GF separator (0.0176 mg h⁻¹), indicating that the use of the hydrophobic separator effectively delays the occurrence of self‐corrosion. In addition, the interfacial HER behavior associated with highly reactive water adversely affects the cycle life of the battery. The mitigating effect of the HfO_2_@GF separator on HER can be quantified by linear scanning voltammetry (LSV). Specifically, Zn||Zn symmetric batteries with different separators are tested at a scan rate of 1 mV s^−1^.^[^ ^56^ ^]^ To avoid interference from Zn deposition on the HER, 2 M Na_2_SO_4_ solution is used as the electrolyte, allowing the current response in the LSV to be directly attributed to the HER.^[^ ^57^ ^]^ As shown in Figure 3b, the hydrophobic HfO_2_@GF separator current density is significantly reduced and delays the HER potential from −0.059 to −0.112 V (15 mA cm^−2^) compared to the hydrophilic GF separator. This indicates that the introduction of the hydrophobic HfO_2_@GF separator effectively alleviates the decomposition of active water molecules and suppresses the occurrence of interfacial HER.

To further reveal the inhibition of interfacial side reactions by the HfO_2_@GF separator, the Zn anode after 50 cycles is characterized by X‐ray photoelectron spectroscopy (XPS). As shown in Figure 3c, the Zn 2p spectrum of the Zn anode with GF separator can be decoupled into two typical signals of Zn: Zn^2+^ (1046.3 eV and 1022.8 eV) and Zn^0^ (1045.2 eV and 1022.1 eV). This implies that significant side reactions have occurred on the surface of the Zn electrode.^[^ ^58^ ^]^ In contrast, the Zn anode with HfO_2_@GF separator shows only one sub‐peak corresponding to Zn^0^, which implies that the side reactions have been effectively suppressed. Similarly, the same conclusion can be obtained in the S 2p spectra (Figure 3d). The Zn electrode cycled with the GF separator shows an obvious SO_4_^2−^ (168.9 eV) signal, implying the generation of the by‐product Zn_4_SO_4_(OH)_6_·xH_2_O (ZSH). In contrast, no significant signal of S is observed in the Zn electrode cycled with the HfO_2_@GF separator, which further validates the superiority of the prepared HfO_2_@GF separator.

Chronoamperometry (CA) is commonly used to provide information related to Zn^2+^ nucleation and growth kinetics.^[^ ^59^ ^]^ As shown in Figure 3e, at a constant overpotential of ‐150 mV, the current density of the Zn anode with GF separator continuously increases over 200 s. This indicates that Zn^2+^ is undergoing continuous 2D diffusion, which tends to lead to disordered deposition of Zn^2+^. In contrast, in the HfO_2_@GF separator, Zn^2+^ rapidly transforms to stable 3D diffusion after a brief (20 s) 2D diffusion and nucleation process. This suggests that the HfO_2_@GF separator effectively promotes the rapid nucleation and growth of Zn^2+^ on the anode surface, which is conducive to forming a dense and flat Zn deposition layer. Figure 3f clearly shows the diffusion process of Zn^2+^ in Zn anodes with different separators. In the GF separator, due to the instability of the electrode/electrolyte interface, Zn^2+^ tends to undergo lateral diffusion along the anode surface and preferentially nucleates at localized tips to minimize surface energy. This results in gradual accumulation and the formation of vertically oriented zinc dendrites. In contrast, in the HfO_2_@GF separator, the electric field distribution is optimized, and the electrode/electrolyte interface stability is enhanced due to significantly improved dielectric properties and hydrophobicity. As a result, the diffusion of Zn^2+^ on the electrode surface is not limited by a specific direction, promoting the formation of denser and more uniform Zn nucleation sites. To gain a deeper understanding of the effect of HfO_2_@GF separators on the initial nucleation behavior during zinc deposition, we performed cyclic voltammetry (CV) tests on Zn||Ti cells with different separators. The nucleation overpotential (NOP) is usually defined as the interval between the B site (reduction potential) and the A site (crossover point).^[^ ^44^ ^]^ As shown in Figure S14 (Supporting Information), the HfO_2_@GF separator shows a reduced NOP (|BB'|≈16 mV), which implies a lowering of the zinc nucleation barrier and helps to promote the rapid ion deposition kinetics.^[^ ^60^, ^61^ ^]^ Moreover, the capacity‐voltage curves of Zn||Cu batteries with different separators at different current densities verified this (Figure S15, Supporting Information). The batteries with HfO_2_@GF separators exhibit low Zn‐shaped core overpotentials at different current densities compared to GF separators. It is worth noting that the HfO_2_@GF separator shows a larger closed‐loop area and higher peak current density, which validates its enhanced role in improving zinc deposition.^[^ ^62^ ^]^

The desolvation process of Zn^2+^ prior to deposition consumes a significant amount of energy, which is often considered a critical parameter in determining the kinetics of zinc deposition. This process is typically measured by the activation energy (E_a_). To accurately assess the effect of the HfO_2_@GF separator on the desolvation process of Zn[H_2_O]_6_^2+^, we test the EIS curves of Zn||Zn batteries with different separators at different temperatures (Figure S16, Supporting Information). The E_a_ is fitted to the charge transfer resistance (R_ct_) obtained at different temperatures based on the Arrhenius equation (Table S2, Supporting Information). As shown in Figure 3g, the E_a_ of the HfO_2_@GF separator is significantly reduced to 33.029 kJ mol^−1^, much lower than the 37.512 kJ mol^−1^ of the GF separator. It indicates that the HfO_2_@GF separator can effectively promote the desolvation process of Zn[H_2_O]_6_^2+^ and enhance the kinetics of the interfacial reaction. This is mainly attributed to the shielding ability of the HfO_2_@GF separator against water molecules. In addition, the Zn^2+^ migration number ($t_{Zn^{2+}}$) is calculated for symmetric batteries with different separator systems (Table S3 and Figure S17, Supporting Information). As shown in Figure 3h, the battery with HfO_2_@GF separator shows a higher $t_{Zn^{2+}}$ (0.729), which is mainly related to the enhanced dielectric properties of the HfO_2_@GF separator. Higher $t_{Zn^{2+}}$ helps to promote charge migration kinetics and reduces the Zn^2+^ concentration gradient in the electrolyte system to reduce the concentration polarization.

To further investigate the regulatory mechanism of the HfO_2_@GF separator on Zn deposition orientation, the adsorption energies of HfO_2_ molecules on different Zn crystal planes are calculated by using density functional theory (DFT). The model of the adsorption of HfO_2_ molecules on different Zn crystal planes is shown in Figure S18 (Supporting Information). As shown in Figure 3i, HfO_2_ shows higher interfacial formation energy (6.65 eV) with Zn (101) crystal planes compared to Zn (100) and Zn (002) crystal planes. This indicates that the HfO_2_@GF separator exhibits stronger binding affinity to the Zn (100) and Zn (002) crystal planes, facilitating Zn^2+^ diffusion into these planes and resulting in a higher deposition rate of Zn^2+^ on both crystal planes. Similarly, since the HfO_2_@GF separator shows the weakest affinity or even repulsive force for the Zn (101) crystal surface, this will result in Zn^2+^ having the lowest deposition rate on the Zn (101) crystal surface. According to Bravais laws, the orientation of crystal planes is determined by the deposition rates of ions on different crystal planes, with the plane having the lowest growth rate ultimately becoming the exposed crystal plane.^[^ ^63^ ^]^ This leads to the preferential exposure of Zn (101) crystalline surfaces due to the HfO_2_@GF separator, resulting in a unique Zn (101) deposition pattern. In addition, the interaction between Zn^2+^ and HfO_2_@GF separator is further investigated using DFT calculation, as shown in Figure 3j. Figures S19 and S20 (Supporting Information) show the computational models of Zn^2+^ on the Zn (101) crystal surface and at different adsorption sites on HfO_2_ crystals, respectively. The results show that the adsorption energy of Zn^2+^ at different sites on the HfO_2_ crystal is higher than that on the Zn (101) crystal plane (‐19.07 eV), indicating that the interaction between Zn^2+^ and the HfO_2_@GF separator is significantly weakened (Figure 3k). This observation is advantageous for mitigating Zn^2+^ aggregation and growth along the separator, thereby improving system stability.

In addition, to fully understand the effect of HfO_2_@GF separator on the ion transport behavior, we further calculated the diffusion energy barriers of Zn^2+^ in HfO_2_ crystals and on the Zn (101) surface by DFT. As shown in Figure 3l, Zn^2+^ shows a low bulk‐phase diffusion energy barrier of 0.98 eV in HfO_2_ crystals, indicating that Zn^2+^ can easily pass through the HfO_2_ layer.^[^ ^64^ ^]^ In addition, the diffusion energy barrier of Zn^2+^ on the Zn (101) surface is 0.43 eV, which is lower than its diffusion energy barrier in HfO_2_. This indicates that after Zn^2+^ passes through the HfO_2_@GF separator, the high diffusion energy barrier impedes its migration, forcing Zn^2+^ to preferentially migrate along the electrode surface rather than penetrate and accumulate toward the separator. This is consistent with the aforementioned adsorption energy calculation results, further verifying the role of the HfO_2_@GF separator in regulating zinc deposition orientation.

### 2.3 The Regulation of Zinc Deposition Morphology by the HfO_2_@GF Separator

**Figure 4:**
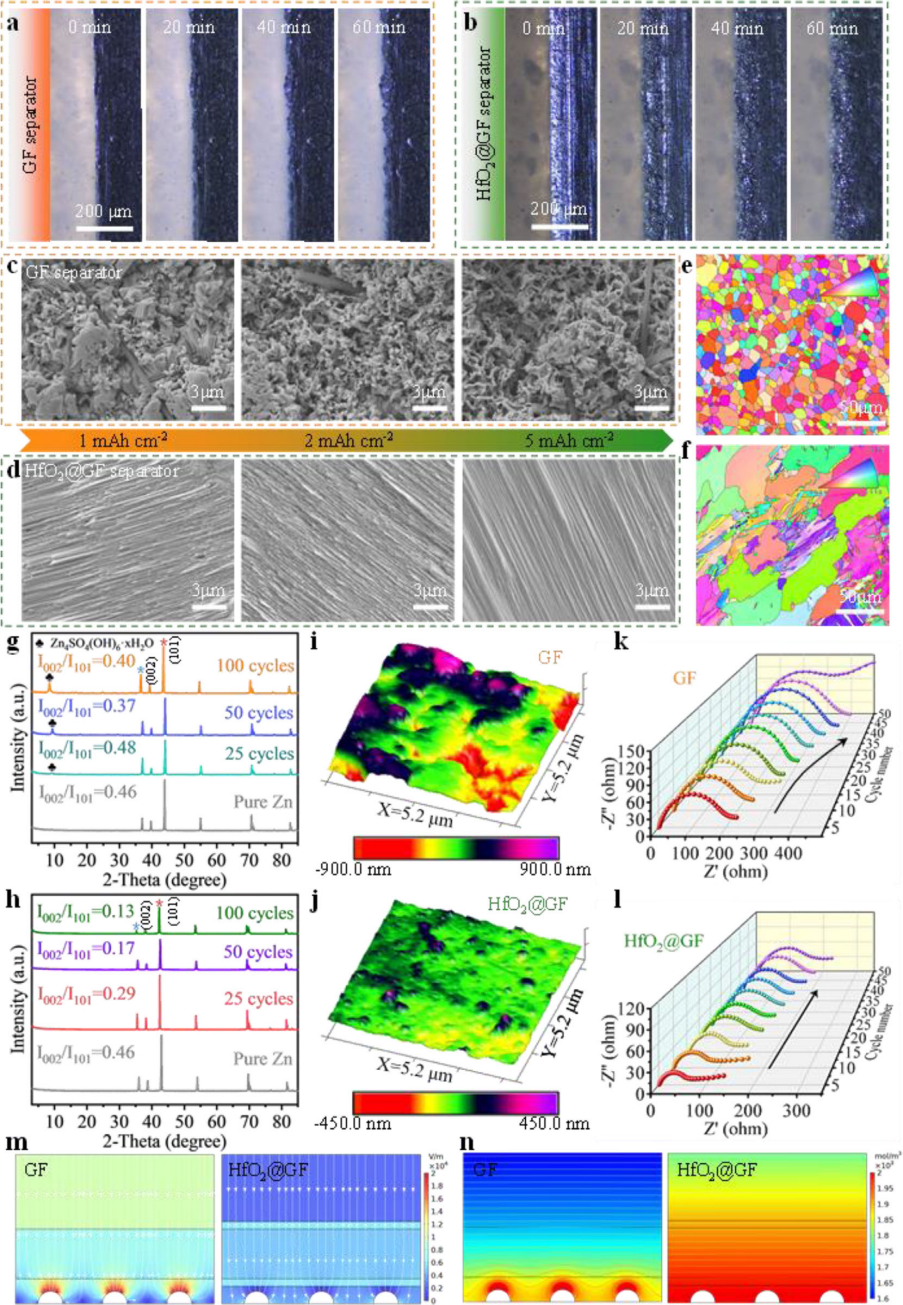
Modulation of zinc deposition morphology by HfO_2_@GF separator. In situ optical microscopy characterization of zinc anodes with a) GF separator and b) HfO_2_@GF separator at a current density of 50 mA cm^−2^. SEM images of Cu foil with c) GF separator and d) HfO_2_@GF separator at different deposition capacities at a current density of 1 mA cm^−2^. EBSD plots of zinc anodes after 50 cycles in e) GF separator and f) HfO_2_@GF separator. XRD patterns of zinc anodes with g) GF separator and h) HfO_2_@GF separator after 25 to 100 cycles. The AFM images of the surface of zinc anodes with i) GF separator and j) HfO_2_@GF separator. XRD patterns of zinc anodes with GF separator and HfO_2_@GF separator after 25 to 100 cycles. Ex situ EIS curves of zinc anodes with k) GF separator and l) HfO_2_@GF separator from the 1^st^ to the 100^th^ cycle. The simulation of m) the interface electric field and n) ion concentration field with different separators.

The evolution of the morphology of the Zn anode surface is an important indicator for describing the behavior of Zn deposition. To obtain direct evidence that the HfO_2_@GF separator can regulate the Zn^2+^ deposition behavior, we employ in situ optical microscopy to monitor the dynamic zinc deposition process on the anode under a current density of 50 mA cm^−2^. As shown in **Figure** **4**a, after 20 min of plating, the surface of the zinc anode with GF separator shows multiple inhomogeneous tiny zinc protrusions. Due to the “tip effect”, these local protrusions gradually develop into large zinc dendrites as the deposition time increases, eventually leading to a rough and uneven electrode surface. In contrast, the zinc anode with the HfO_2_@GF separator exhibits a dense and smooth zinc deposition morphology throughout the entire plating process, without any visible zinc protrusions (Figure 4b).

To further verify the modulation of Zn deposition behavior and the inhibition of interfacial side reactions by the HfO_2_@GF separator, we disassemble the symmetric battery and perform SEM observation of the micro‐morphology of the circulating Zn electrode. As shown in Figure S21 (Supporting Information), the Zn electrode with GF separator shows a rough surface accompanied by a large number of porous and loose dendritic Zn protrusions. Notably, these randomly distributed zinc protrusions will exacerbate the tendency for parasitic reactions to occur on the electrode surface. In contrast, the Zn anode under the protection of the HfO_2_@GF separator shows a flat and dense surface morphology, with Zn^2+^ growing along the Zn (101) crystal surface in a layer‐by‐layer orderly manner. In addition, the EDS spectra show severe surface corrosion of the circulating Zn metal due to the excessive hydrophilicity of the GF separator. The high content of elemental O (32.78%) and S (5.15%) indicates that a large amount of flaky by‐products are generated on the electrode surface (Figure S22, Supporting Information). It is noteworthy that the surface elements of the Zn electrode circulated with the HfO_2_@GF separator are uniformly distributed, and the ZSH‐related signals are hardly detected (Figure S23, Supporting Information).

The SEM images of zinc deposition on Cu foil at different deposition capacities further reveal the microstructure of the zinc electro‐deposition layer. As shown in Figure 4c, with the GF separator, some uneven zinc deposits randomly appear at 1 mAh cm^−2^. As the deposition capacity increases, the “tip effect” gradually intensifies, eventually leading to loose and porous zinc electro‐deposition, which causes the separator to puncture. In contrast, the HfO_2_@GF separator provides a more uniform and smooth electro‐deposition layer. As deposition progresses, it exhibits a distinct Zn (101) crystal plane orientation (Figure 4d). Electron Backscatter Diffraction (EBSD) further characterizes the crystallographic orientation of the cycled zinc anode.^[^ ^65^ ^]^ As shown in Figure 4e, the zinc anode cycled for 50 cycles with the GF separator displays a large number of disordered color blocks, revealing randomly oriented zinc deposition. In contrast, the zinc anode cycled with the HfO_2_@GF separator shows a distribution predominantly in the green series, which represents the (101) crystal plane of metallic Zn.^[^ ^66^ ^]^ This indicates that the introduction of the HfO_2_@GF separator helps to promote the exposure of the Zn (101) crystal plane (Figure 4f).

To investigate the compositional evolution of the electrode surface during the cycling process, XRD is employed to analyze the crystalline surface texture changes of Zn electrodes with different separator configurations. As shown in Figure S24 (Supporting Information), metallic Zn exhibits a hexagonal close‐packed (hcp) structure with dominant crystal planes at (002), (100), and (101).^[^ ^67^, ^68^ ^]^ Due to variations in grain orientation, different crystal planes exhibit distinct deposition behaviors. As shown in Figure 4g, compared to the original Zn anode, the intensity ratio of the (002) diffraction peak to the (101) diffraction peak (denoted as I_002_/I_101_) for the Zn anode based with the GF separator exhibits an irregular variation trend after different cycle numbers. It is shown that the deposition pattern of Zn^2+^ on the zinc anode during the cycling process exhibits a randomly varying deposition orientation. In contrast, the I_002_/I_101_ value of the Zn anode with the HfO_2_@GF separator consistently decreases over 100 cycles (Figure 4h), indicating that the HfO_2_@GF separator guides preferential deposition of Zn^2+^ along the Zn (101) crystal plane. In addition, compared to the HfO_2_@GF separator, the XRD pattern of the Zn anode with the GF separator shows a distinct diffraction peak at 7.68°, corresponding to the formation of the by‐product ZSH. This is primarily because the HfO_2_@GF separator effectively removes active water and SO_4_^2−^ from the Zn anode surface, thus mitigating side reactions.

In addition, we further characterize the circulating zinc foils by grazing incidence X‐ray diffraction (GIXRD), which is a powerful tool to study the film weave and orientation anisotropy.^[^ ^69^ ^]^ As shown in Figure S25 (Supporting Information), the value of I_002_/I_101_ for the zinc anode cycled with the HfO_2_@GF separator is significantly lower than that of the GF separator. This further verifies that the HfO_2_@GF separator helps to form a unique Zn (101) crystal plane. Atomic force microscopy (AFM) is commonly used to characterize the surface roughness of Zn foils after cycling. A large number of sharp protrusions are observed on the Zn electrodes after 50 cycles with the GF separator, presenting an uneven and rough surface (Figure 4i). However, benefiting from the directional dipole‐induced spontaneous polarization electric field, Zn metal equipped with the HfO_2_@GF separator exhibits a smoother and flatter surface (Figure 4j). This improved surface morphology is conducive to promoting homogeneous zinc plating/stripping behavior.

Ex situ EIS is commonly used to characterize the ion transport kinetics during the zinc deposition process. Figure 4k,l display the EIS of the Zn||GF||Zn and Zn||HfO_2_@GF||Zn batteries at different cycle numbers, respectively. The results indicate that the GF separator exhibits irregular and progressively increasing interfacial impedance, revealing an unstable electrode/electrolyte interface and significantly deteriorated ion transport kinetics.^[^ ^70^ ^]^ This deteriorated interfacial performance is likely caused by the passivation of the anode due to the formation of randomly oriented Zn dendrites and non‐conductive by‐products. These factors lead to restricted ion transport and result in inhomogeneous Zn^2+^ plating/stripping behavior. This deteriorated interfacial performance may be attributed to the passivation of the anode, caused by the formation of randomly oriented Zn dendrites and non‐conductive by‐products. These factors lead to restricted ion transport and result in inhomogeneous Zn^2+^ plating/stripping behavior. In contrast, the battery based on the HfO_2_@GF separator shows a relatively stable interfacial transfer impedance. This indicates that the ion transfer kinetics at the solid‐liquid interface is improved, which is conducive to the construction of a uniformly distributed Zn^2+^ flux. The built‐in polarized electric field induced by the HfO_2_@GF separator can provide uniform electric and ionic fields at the interface, thus promoting the smooth deposition of Zn^2+^. Additionally, the separator possesses unique hydrophobicity, effectively reducing water‐induced parasitic reactions. These properties lead to enhanced reversibility and long‐term cycling performance of the battery.

To further investigate the role of the HfO_2_@GF separator in modulating Zn^2+^ deposition and nucleation behaviors, finite element simulations are conducted using COMSOL Multiphysics. These simulations help elucidate the evolution of interfacial electric and concentration fields. Simplified geometrical models of Zn||Zn symmetric batteries with GF separator and HfO_2_@GF separator are shown in Figure S26 (Supporting Information). In addition, we set the dielectric constants of the different separators to simulate the effect caused by the dielectric constants. As shown in Figure 4m,n, in the GF separator, localized irregular protrusions on the surface of commercial zinc foil lead to an uneven distribution of the electric field strength and Zn^2+^ flux. This uneven distribution triggers inhomogeneous zinc nucleation sites. During subsequent deposition, the relatively concentrated electric field at these protrusions will lead to a continuous accumulation of Zn^2+^, which will exacerbate the “tip effect”. This positive feedback process ultimately results in uncontrollable vertical dendrite formation, leading to premature battery failure. Encouragingly, the introduction of HfO_2_@GF separator effectively avoids this self‐accelerating “tip effect.” The electric field at the protrusions on the anode surface is greatly weakened and produces a uniformly distributed spatial interfacial electric field, which induces the formation of uniform nucleation sites. This favors the formation of uniform dendrite‐free deposition morphology. Furthermore, the suppression of the “tip effect” leads to a reduced Zn^2+^ concentration gradient and enables a stable ionic flow, which is conducive to minimizing concentration polarization. These results strongly confirm that the HfO_2_@GF separator can achieve a uniform distribution of Zn^2+^ flux, which contributes to the realization of dendrite‐free zinc deposition. The current density distribution at the interface also proves this (Figure S27, Supporting Information).

### 2.4 Electrochemical Performance of Half‐batteries with HfO_2_@GF Separator

**Figure 5:**
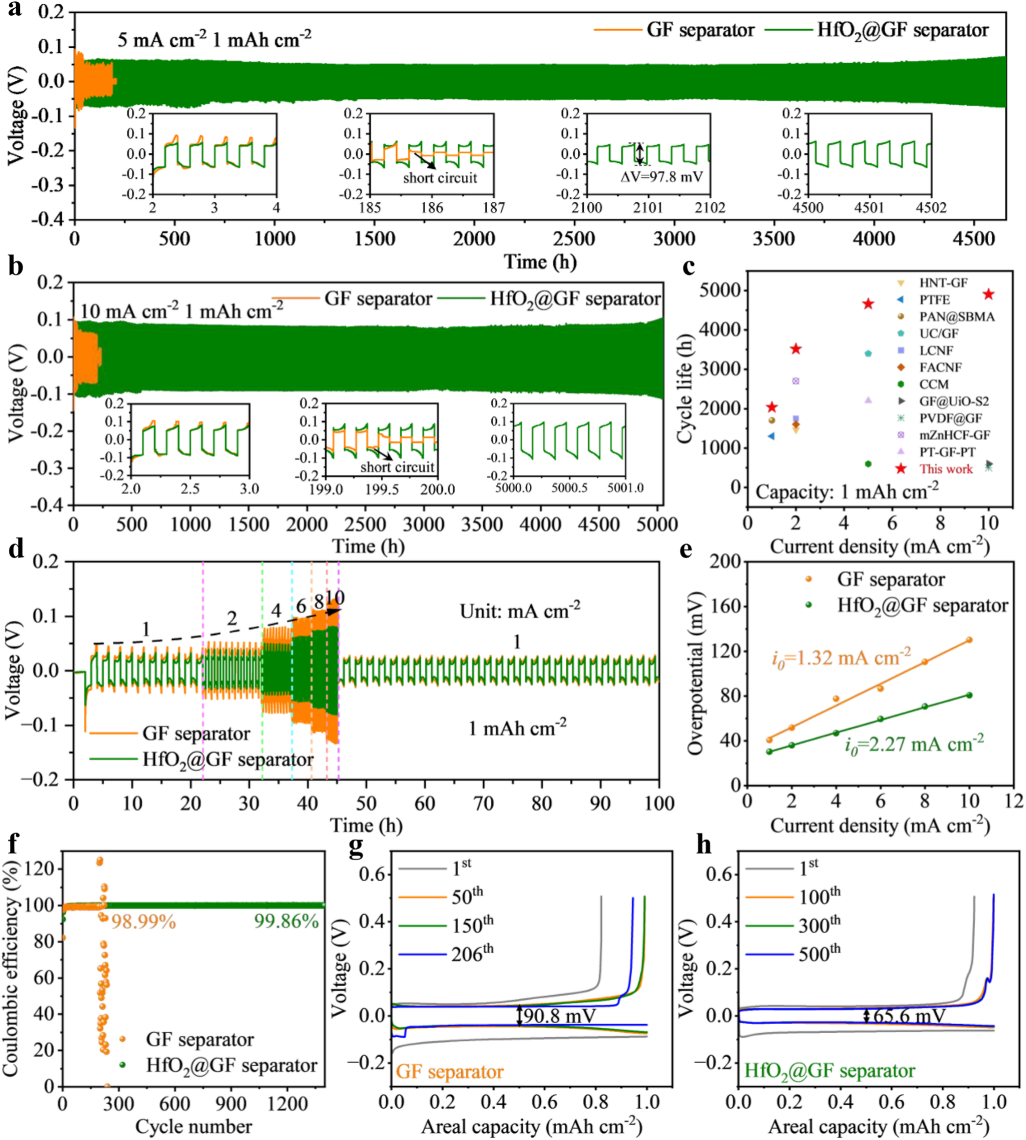
Electrochemical performance of half‐batteries. Long‐cycle performance of Zn||Zn symmetric batteries with different separators at a) 5 mA cm^−2^, 1 mAh cm^−2^ and b)10 mA cm^−2^, 1 mAh cm^−2^. c) Comparison of the cycling life of Zn||Zn symmetric batteries with HfO_2_@GF separator and recently reported separator modification works. d) The rate performance of batteries with different separators at a fixed areal capacity of 1 mAh cm^−2^. e) The fitting of the exchange current density for Zn||Zn symmetric batteries with different separators. f) Coulombic efficiency of Zn||Cu asymmetric batteries with different separators at a current density of 5 mA cm^−2^ and a capacity of 1 mAh cm^−2^, with a cutoff voltage of 0.5 V. The capacity‐voltage curve of Zn||Cu batteries with g) GF separator and h) HfO_2_@GF separator.

To confirm the positive effect of the HfO_2_@GF separator on the electrochemical performance enhancement of the zinc anode, we prepare Zn||Zn symmetric batteries based on different separators. The constant‐current long‐cycle tests are conducted under different test conditions. Batteries with HfO_2_@GF separators show impressive long‐term cycling stability. As shown in **Figure** **5**a, the Zn||Zn symmetric battery with HfO_2_@GF separator can be operated stably for 4660 h with a stable polarization voltage at a current density of 5 mA cm^−2^ and an areal capacity of 1 mAh cm^−2^. In contrast, due to side reactions such as uneven deposition and corrosion, batteries with GF separators show high instability in the early stages of cycling, accompanied by significant voltage lag. As cycling progresses, the by‐product layer on the electrode surface gradually cracks and detaches, leading to a reduction in voltage lag. However, it ultimately results in a short circuit after only 185 h of operation due to dendrites piercing the separator. In contrast, batteries with HfO_2_@GF separators effectively suppress side reactions and avoid voltage fluctuations, demonstrating improved interface stability. The corresponding zoom curves show the zinc plating/stripping profiles for symmetric batteries based on different separators at different cycling moments. More surprisingly, the Zn||Zn symmetric battery based on the HfO_2_@GF separator can still achieve an ultra‐long cycle life of 5050 h at a higher current density of 10 mA cm^−2^ (Figure 5b). This is 25 times longer than the Zn||Zn battery based on the GF separator, which only lasts for 199 h, demonstrating excellent electrochemical stability. In addition, the battery with HfO_2_@GF separator can survive for 2035 and 3513 h at current densities of 1 and 2 mA cm^−2^, respectively. Its performance is significantly better than that of the symmetric batteries based on the GF separator (Figure S28, Supporting Information). This strongly confirms the positive role of the HfO_2_@GF separator in inducing uniform zinc plating/stripping behavior and suppressing side reactions. Compared to recently reported work on separator modification, batteries with HfO_2_@GF separator offer competitive advantages in terms of current density and cycle life (Figure 5c; Table S4, Supporting Information).

To obtain the total overpotential (*η*) at different current densities, the rate performance of Zn||Zn symmetric batteries with different separators is evaluated. The current densities range from 1 to 10 mA cm^−2^, with a fixed areal capacity of 1 mAh cm^−2^ (Figure 5d). The results show that a battery with HfO_2_@GF separator can achieve stable cycling at different current densities, exhibiting more excellent rate performance and lower voltage hysteresis. Meanwhile, compared to the GF separator (1.32 mA cm^−2^), the battery based on the HfO_2_@GF separator can provide a higher exchange current density of 2.27 mA cm^−2^ (Figure 5e; Table S5, Supporting Information). This indicates that the HfO_2_@GF separator effectively enhances the Zn^2+^ deposition kinetics.^[^ ^63^ ^]^ Specifically, higher exchange current densities contribute to a larger critical nucleation radius during the initial nucleation process, resulting in a flat and dense zinc deposition.^[^ ^15^ ^]^

To further verify the significant contribution of the high dielectric constant material HfO_2_ to cycling performance, we fabricate modified GF separators coated with other low dielectric constant materials ($\varepsilon_{SiO_{2}}$≈1.56, *ε*_ZnO_≈2.5–3.5). These are referred to as SiO_2_@GF separator and ZnO@GF separator, respectively. Constant current charge/discharge tests are conducted based on symmetric batteries. As expected, the HfO_2_@GF separator with high dielectric constant material can significantly extend the cycle life of the battery. Specifically, the symmetric batteries based on SiO_2_@GF separator and ZnO@GF separator experience short circuits after 1003 and 674 h, respectively. These cycle lives are much lower than that of the HfO_2_@GF separator, demonstrating poor cycle stability (Figure S29, Supporting Information). The reason for this dilemma may be that the separator with a low dielectric constant coating is unable to provide a uniform interface electric field and ion flux. In contrast, a separator with a high dielectric constant coating can effectively homogenize the electric field and ion flux at the interface during cycling. This helps repel harmful mobile anions, minimize concentration polarization, and provide a regular and orderly Zn^2+^ migration path, thus enabling the long‐life cycling of AZMBs.

Coulombic efficiency (CE) is an important parameter for assessing the reversibility of the plating/stripping process. We assemble Zn||Cu half‐batteries with different separators and compare their CE under discharge/charge test conditions at a current density of 5 mA cm^−2^ and an areal capacity of 1 mAh cm^−2^. As shown in Figure 5f, Zn||HfO_2_@GF||Cu maintains an average CE of ≈99.86% over 1400 cycles. However, the battery with the GF separator shows significant fluctuations in CE after 197 cycles, with an average CE of only 98.99% over the 197 cycles. This unstable plating/peeling process may originate from the inhomogeneous zinc deposition behavior and the continuous accumulation of by‐products. Meanwhile, the Zn||Cu battery with HfO_2_@GF separator shows stable and almost overlapping voltage profiles at different cycle numbers compared with the GF separator (Figure 5g,h). It is verified that the HfO_2_@GF separator can effectively enhance the reversibility of the Zn^2+^ plating/stripping process during cycling.

### 2.5 Practical Application of Full Batteries with HfO_2_@GF Separator

To further explore the potential of HfO_2_@GF separators for practical applications in AZMBs, full batteries are assembled to investigate their energy storage capacity. The cathode active material Zn_0.25_V_2_O_5_ (ZVO) is synthesized by a simple hydrothermal method. It can be observed by SEM that ZVO exhibits a typical nanorod‐like structure (Figure S30, Supporting Information). The Zn||HfO_2_@GF||ZVO full battery with a mass loading of 4.3 mg cm^−2^ shows excellent performance. As shown in Figure S31 (Supporting Information), the Zn||ZVO battery with GF separator shows complete capacity decay after 350 cycles. However, the Zn||HfO_2_@GF||ZVO battery still maintains a capacity of 150.2 mAh g^−1^ after 1000 cycles at a rate of 10C, which shows excellent cycling stability. In addition, compared with the GF separator, Zn||ZVO battery based on the HfO_2_@GF separator can provide more excellent rate performance (Figure S32, Supporting Information). It can provide discharge specific capacities of 316.5, 286.0, 255.6, 233.5, 215.6, and 197.6 mAh g^−1^ at different rates, respectively. These results effectively demonstrate the durability of the HfO_2_@GF separator.

**Figure 6:**
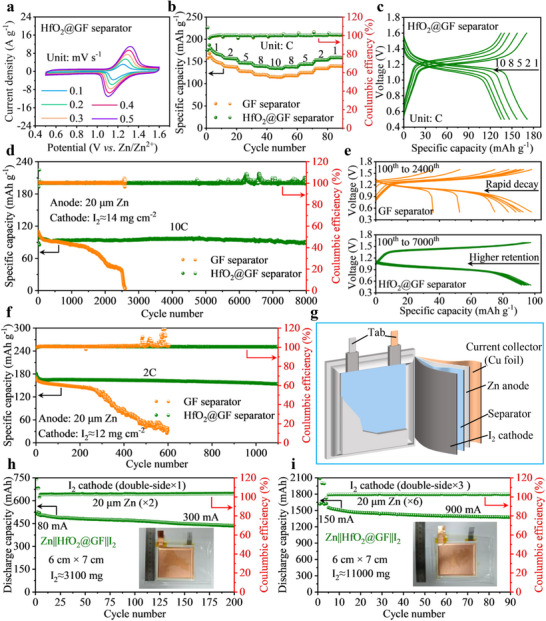
Electrochemical performance of full batteries. a) CV curves of Zn||I_2_ battery with HfO_2_@GF separator at different scan rates. b) The rate performance of Zn||I_2_ batteries with different separators. c) Capacity‐voltage curves of the battery with HfO_2_@GF separator at different rates. d) The long‐cycle performance of Zn||I_2_ full batteries with different separators at 10C. e) Capacity‐voltage curves of batteries with different separators at different cycles. f) The long‐cycle performance of Zn||I_2_ full batteries with different separators at 2C. g) The schematic diagram of the Zn||I₂ pouch battery. h) The cycling performance of the single‐layer Zn||I_2_ pouch battery with HfO_2_@GF separator at 300 mA, with a size of 6 cm × 7 cm. i) The cycling performance of the three‐layer Zn||I_2_ pouch battery with HfO_2_@GF separator at 900 mA, with a size of 6 cm × 7 cm.

To fully explore the reliability of the HfO_2_@GF separator for practical applications, a thin zinc foil (20 µm) is further coupled with a highly loaded I_2_ positive electrode. The CV curves show a high degree of overlap at a scan rate of 1 mV s^−1^ for different scanning turns, indicating a highly reversible behavior (Figure S33, Supporting Information). In addition, there is no significant difference in the redox peaks of the CV curves of batteries with different separators, indicating that the introduction of the HfO_2_@GF separator does not affect the redox reaction of the I_2_ cathode. To further investigate the electrochemical reaction kinetics of Zn||I_2_ full batteries with different separators, further CV tests are performed on the full batteries at scan rates ranging from 1 to 5 mV s^−1^ (**Figure** **6**a; Figure S34, Supporting Information). The electrochemical contribution of the electrodes is qualitatively analyzed by the correlation between the measured response current (i) and the scan rate (v), given by the following equation: log (i) =  a + blog(v).^[^ ^71^ ^]^ In general, when the b value is close to 0.5, the dominant controlled process is ion diffusion, in contrast, when b is close to 1, it indicates a capacitive controlled process.^[^ ^12^, ^72^ ^]^ The calculated b‐values corresponding to the oxidation and reduction peaks are 0.785 and 0.677, respectively (Figure S35, Supporting Information). This indicates that the electrochemical kinetics are synergistically dominated by surface‐controlled and diffusion‐controlled behaviors.

Subsequently, the rate performance of the Zn||I_2_ batteries is evaluated in the rate range of 1 to 10C, as shown in Figure 6b. The results show that the HfO_2_@GF separator‐based battery delivers higher discharge specific capacity than the GF separator at different rates and good capacity recovery. This further confirms the high reversibility and fast ion transport capability of the HfO2@GF separator. The voltage platforms shown in Figures 6c and S36 (Supporting Information) further validate this improved rate capability. As the rate increases from 1 to 10C, the Zn||HfO_2_@GF||I_2_ battery demonstrates a higher discharge specific capacity. This is attributed to the fact that the spontaneous polarization electric field generated by HfO_2_ during charging and discharging accelerates the migration of Zn^2+^, resulting in enhanced rate performance. Further, the long‐term cycling performance of Zn||I_2_ full batteries based on different separators is investigated, as shown in Figure 6d. Impressively, the full battery with the HfO_2_@GF separator shows surprising long‐term tolerance. Specifically, the Zn||HfO_2_@GF||I_2_ battery still has a capacity of 88.8 mAh g^−1^ after 8000 cycles at a rate of 10C, showing remarkable capacity retention (87.75%). In contrast, the Zn||GF||I_2_ battery shows rapid capacity decay and quickly fails after only 2600 cycles. In addition, the capacity‐voltage curves of the full battery at different cycle numbers further indicate that the battery with the HfO_2_@GF separator possesses relatively stable long‐term cycling stability (Figure 6e). The significant improvement in cycling stability is primarily due to the HfO_2_@GF separator, which ensures uniform and reversible zinc plating/stripping behavior and effectively minimizes side reactions. Furthermore, at a rate of 2C, the Zn||HfO_2_@GF||I_2_ battery demonstrates superior cycling stability compared to the Zn||GF||I_2_ battery, retaining 84.91% of its initial capacity after 1100 cycles (Figure 6f). In addition, the self‐discharge of full batteries is also a challenging issue. Firstly, the battery is charged to a 100% state of charge (SOC) at a rate of 10C (battery voltage is 1.5 V), then it is left to stand for 24 h. Finally, the SOC of the battery is reduced to 0% at the same rate (battery voltage is 0.5 V). As shown in Figure S37 (Supporting Information), benefit from the outstanding side reaction suppression ability of the HfO_2_@GF separator, the Zn||HfO_2_@GF||I_2_ full battery retains 86.04% of its initial capacity after long‐term self‐discharge, which is higher than the GF separator (75.35%).

The applicability of the HfO_2_@GF separator in pouch batteries is tested by assembling 6 cm × 7 cm pouch batteries (Figure 6f). The cycling performance of the pouch batteries is achieved by applying pressure using a fixture during operation. As shown in Figure 6g, with a 74 mg cm^−2^ I_2_ cathode mass loading, the single‐layer Zn||HfO_2_@GF||I_2_ pouch battery achieves a maximum discharge capacity of 524.77 mAh and demonstrates good cycling stability. And it still retains 84.69% of its original capacity after 200 cycles at 300 mA. To further illustrate the practical application possibility of the HfO_2_@GF separator, we fabricate a multilayer stacked structure Zn||I_2_ pouch battery. Specifically, it mainly consists of 6 pieces of 20 µm Zn anodes, 6 pieces of separators, and 3 double‐sided I_2_ cathodes. Notably, the three‐layer pouch battery with HfO_2_@GF separators is capable of providing Ah‐level discharge capacity and demonstrates good long‐term endurance. The multi‐layer Zn||I_2_ pouch battery provides an initial discharge capacity of 1.64 Ah at a current of 900 mA after 90 cycles, and it still retains 83.77% of its capacity (Figure 6h). These results highlight the practical application potential of the HfO_2_@GF separator in AZMBs.

## 3 Conclusion

In summary, we present an effective strategy for separator modification by spin‐coating hydrophobic PVDF and high‐dielectric HfO_2_ onto commercial GF separators to enable extended cycling of AZMBs. The prepared HfO_2_@GF separator shows significantly enhanced dielectric properties and superhydrophobicity. Under the influence of the external electric field, HfO_2_ can spontaneously generate an enhanced internal electric field via the Maxwell polarization effect.

This effectively homogenizes the electric field and ion flux distribution at the interface, enabling ordered and rapid Zn^2+^ transport while electrostatically repelling the accumulation of anions near the anode. Moreover, the exceptional hydrophobicity of the separator significantly reduces water‐induced corrosion and HER. Notably, the HfO_2_@GF separator exhibits weaker chemical interaction with the Zn (101) crystal surface, which facilitates the exposure of an atypical Zn (101) crystalline texture for dense and orderly dendrite‐free Zn deposition. Therefore, a symmetric battery with HfO_2_@GF separator achieves an ultra‐long cycle life of 4660 h (more than 6 months at 5 mA cm^−2^ and 1 mAh cm^−2^). When the current density is increased to 10 mA cm^−2^, the symmetric battery still operates stably for 5050 h, showing significantly enhanced cycle stability. Interestingly, the 20 µm Zn||I_2_ full battery with HfO_2_@GF separator still has a high capacity retention (87.75%) after 8000 cycles at 10C. More importantly, the single‐layer 20 µm Zn||I_2_ pouch battery assembled with the HfO_2_@GF separator can achieve 200 cycles at 300 mA while retaining 84.69% of its initial capacity. Furthermore, the multi‐layer pouch battery provides a high discharge capacity of 1.64 Ah, maintaining a good capacity retention of 83.77% after 90 cycles. This work highlights the regulation of ion behavior by the interfacial electric field and crystal orientation, providing new insight for achieving long‐cycle life in AZMBs.

## Conflict of Interest

The authors declare no conflict of interest.

## Supporting information



Supporting Information

## Data Availability

The data that support the findings of this study are available from the corresponding author upon reasonable request.
