# Supplementary material for: Synergistic Enhancement Effect of Maxwell Polarization and Preferential Exposure of Zn (101) Plane toward Superhydrophobic Separator for Ah‐Level Zinc Metal Pouch Batteries
Source: Adv Sci (Weinh). 2025 Jul 30;12(35):e06035. doi: 10.1002/advs.202506035 (PMC12462938; doi:10.1002/advs.202506035)
Supplement: Supplementary file 1 — Supporting Information [file ADVS-12-e06035-s001.docx]

Supporting Information

**Synergistic Enhancement Effect of Maxwell Polarization and Preferential Exposure of Zn (101) Plane Towards Superhydrophobic Separator for Ah-Level Zinc Metal Pouch Batteries**

Xinyao Yuan^1#^, Di Zhang^1#^, Hongfei Lu^1^, Yuhang Song^1^, Zhiyi Du^1^, Minjie Song^1^, Nawei Lyu^1^, Yang Jin^1*^

Miss X. Yuan, Dr. D. Zhang, Dr. H. Lu, Dr. Y. Song, Miss Z. Du, Miss M. Song, Dr. N. Lyu, Prof. Y. Jin

Research Center of Grid Energy Storage and Battery Application, School of Electrical and Information

Engineering, Zhengzhou University, Zhengzhou, Henan 450001, China

E-mail: yuanxinyao8782@163.com, zhangdi3512@163.com, luhfff@126.com, yhsong@gs.zzu.edu.cn, dzy200101716@gs.zzu.edu.cn, songminjie@gs.zzu.edu.cn, naweilyu@zzu.edu.cn, yangjin@zzu.edu.cn

**Experimental section**

**Materials:** Glass fiber separator (GF/C, 110 mm diameter, 100 circles, No. 1822-110) was purchased from Whatman. HfO_2_ (99.99%, 50-100nm) was purchased from Adamas. SiO_2_, ZnO and ZnI2 (AR, ≥98%) were purchased from Macklin. (CH_3_COO)_2_Zn·2H_2_O, ZnSO_4_·7H_2_O (AR), ethyl alcohol (AR), and Zn foil (200 μm thick, 99.9%) were bought from Sinopharm Chemical Reagent. Zn foil (20 μm thick, 99.9%) were bought from Haiyuan Technology Metal. Cu foil, Ti network, Acetylene black and Activated carbon (AC, 200 mesh) were purchased from Taobao. Ti foil (10 μm thick, 99.999%) was bought from Haiyuan Research Metal. Polyvinylidene fluoride (PVDF, 99%) powder was purchased from Taiyuan Lizhiyuan Company. Polytetrafluoroethylene (PTFE) dispersion (60 wt% PTFE aqueous solution) was bought from Lizhiyuan Technology. V_2_O_5_ powder (AR) was bought from Enox Reagent. Acetylene black was bought from Cyberchem Materials. Red ink was bought from Zhejiang Hero Cultural Products Co., LTD. N-Methyl pyrrolidone (NMP, 99.5%) was bought from the Aladdin.

**Preparation of HfO_2_@GF, SiO_2_@GF and ZnO@GF separator:** Weigh 450 mg of HfO_2_ and 50 mg of PVDF using an electronic balance, and place them into a glass bottle. Add 1650 µL of NMP and stir for 12 hours until the mixture is uniform. Using a spin coater, evenly spin coat the prepared slurry onto one side of the GF separator. Set the spin-coating speed to 4000 rpm and the time to 60 seconds. Then, vacuum dry the spin-coated separator at 60°C for 12 hours. Afterward, repeat the spin-coating process on the other side of the GF separator using the same method. Similarly, prepare the SiO_2_@GF separator and ZnO@GF separator using the same method. Finally, cut the separators into circular discs with a diameter of Φ19 mm for later use.

**Preparation of aqueous electrolyte:** Preparation of 100 mL 2 mol L^-1^ ZnSO_4_ electrolyte: The 2 mol L^-1^ ZnSO_4_ electrolyte was prepared using ZnSO_4_·7H_2_O and deionized water. 57.512 g of ZnSO_4_·7H_2_O was dissolved in 74.8 mL of deionized water and stirred for 6 hours to form 100 mL of 2 mol L^-1^ ZnSO_4_ electrolytes.

**Preparation of Zn_0.25_V_2_O_5_·nH_2_O (ZnVO) nanoribbons and ZnVO cathode.** First, 2 mmol of V_2_O_5_ was dispersed in a 50 mL water/acetone (15:1) mixture with 1.3 mmol of CH_3_COO)_2_Zn and stirred for 2 hours. The solution was then transferred to a sealed PTFE and kept at 200 °C for 72 hours. Then the obtained products were washed with deionized water, centrifuged, and finally dried at 60℃ for 24 hours to obtain Zn_0.25_V_2_O_5_. The product was ground in an agate mortar for no less than 20 minutes before use. The cathode slurry was prepared as follows: first, NMP (4 mL) was added to a glass vial, then PVDF (100 mg) was added and stirred for 6 hours. Then acetylene black (200 mg) and ZVO (700 mg) were added in a mortar. It was ground for 20 min and then poured into the glass jar and stirred for 12 hours. The mixed slurry was then poured onto a carbon felt and spread evenly using the 200 μm scraper and kept at 80 ℃ for 6 h to obtain the ZVO cathode. The loading mass of ZVO was about 4.3 cm^-2^.

**Preparation of I_2_ cathode:** Activated carbon (AC), acetylene black, and polytetrafluoroethylene (PTFE) binder were mixed in a mass ratio of 8:2:1. The mixture was then rolled onto a Ti mesh current collector to obtain an activated carbon film with a size of 6 cm × 7 cm, which was used as the host material for the active substance I_2_. The 0.5 M ZnI_2_ aqueous solution was electroplated under constant current (1000 mA) for 18 minutes to obtain an I_2_ cathode supported by AC. After I_2_ deposition, the composite electrode was washed with ultrapure water, dried, and used as the cathode for the Zn||I_2_ pouch battery. The thickness of the activated carbon films (excluding Ti mesh) for the coin cell and pouch battery was 0.4 mm and 1 mm, respectively. Additionally, the electroplating current for the I_2_ cathode in the pouch battery is set to 2000 mA, and the electrodeposition is carried out for 39 minutes.

**Assembly of coin cells:** All button cell batteries use CR2025 as the battery case. All battery cases, gaskets and springs were ultrasonically cleaned with ethanol and completely dried before battery assembling. The zinc flakes are sanded with sandpaper to remove the surface oil and oxide layer before use and cleaned with alcohol. From bottom to top, the assembly sequence of Zn||Zn symmetric cell is: cathode shell, Φ12 zinc foil with 200 μm thickness, Φ19 mm separator, Φ12 zinc foil with 200 μm thickness, Φ16 mm gasket with 0.5 mm thick, 1.1 mm spring,and anode shell. The assembly sequence of Zn||Cu half cells was as follows: cathode shell, 100 μm Cu foil, Φ19 mm separator, Φ12 mm Zn foil with 200 μm thickness, Φ16 mm gasket with 0.5 mm thick, 1.1 mm spring, and anode shell. The Zn||Ti half cells were assembled as follows: cathode shell, 10 μm Ti foil, Φ19 mm separator, Φ12 Zn foil with 200 μm thickness, Φ16 mm gasket with 1 mm thick, 1.1 mm spring, and anode shell. The assembly sequence of Zn||ZVO full cell is as follows: cathode shell, Φ11 mm cathode foil, Φ19 mm separator, Φ12 zinc foil with 200 μm thickness, 1 mm gasket, 1.1 mm spring, and anode shell. The assembly sequence of Zn||I_2_ full cell is as follows: cathode shell, Φ11 mm cathode foil, Φ19 mm separator, Φ12 zinc foil with 20 μm thickness, 0.5 mm gasket, 1.1 mm spring, and anode shell. The volume of electrolyte was ≈100 μL per cell. The cell sealing pressure is greater than 50 kg cm^-2^. Before testing, the battery was left at room temperature for about 2 hours.

**Assembly of pouch cells:** All pouch cells are assembled in an air environment at room temperature. The assembly sequence of the pouch cell from bottom to top is as follows: Cu current collector, Zn anode, separator, I_2_ cathode, separator, Zn anode, Cu current collector. For the single-layer Zn||I_2_ pouch cell, two pieces of Zn foil with a thickness of 20 μm and purity greater than 99.9% are used as electrodes, along with two pieces of Cu current collector, two pieces of separator, and one double-sided I_2_ cathode. The large-scale Ah-level multi-layer Zn||I_2_ pouch cell is obtained by repeatedly stacking Zn anodes, separators, and I_2_ cathodes. Specifically, the three-layer Zn||I_2_ pouch cell uses six pieces of Zn foil with a thickness of 20 μm and purity greater than 99.9% as electrodes, four pieces of Cu current collector, six pieces of separator, and three I_2_ cathodes. The dimensions of the electrodes and separators are 6 cm×7 cm and 7.5 cm×8 cm, respectively. Cu foil and Ti mesh are used as current collectors for the anode and cathode, respectively. The width of the tabs is 1 cm. The electrolyte is a 2 M ZnSO_4_ solution containing 0.1 M BMMF. The amount of electrolyte added to the pouch cell is calculated based on the separator area, which is 95 μL cm^-2^. The pouch cells are packaged using transparent vacuum bags. The sealing of the pouch cells is done using a vacuum pre-sealing machine from Hefei Kecing Materials Technology Co., Ltd. The packaging temperature for the transparent vacuum bags is 145 ℃.

**Electrochemical measurements.** All coin cells use the CR2025 as the battery casing. The electrolyte is 2 M ZnSO_4_ solution, and the electrolyte volume in the battery test is 100 μL. Linear polarization (LSV) curves, Tafel curves, chronoamperometry (CA) curves, electrochemical impedance spectroscopy (EIS, frequency range from 0.02 Hz to 100 kHz), ionic conductivity, and cyclic voltammetry (CV) curves are performed on a Bio-Logic VSP five-channel electrochemical workstation. To avoid zinc deposition, 2 M Na_2_SO_4_ solution is used as the aqueous electrolyte in Zn||Zn symmetric cells with different separators, and LSV tests are conducted in the range of -0.25 V to 0.25 V with a scan rate of 1 mV s^-1^. Tafel curves of Zn||Zn batteries with different separators are obtained using 2 M ZnSO_4_ solution as the electrolyte in the range of -0.15 V to 0.15 V at a scan rate of 1 mV s^-1^. The corrosion potential and corrosion current density are obtained through linear fitting of the Tafel plot. The initial potential for the CA test of Zn||Zn symmetric cells is -150 mV, and the test duration is 600 s. The CV test of Zn||I_2_ full cells is conducted in the voltage range of 0.5 V to 1.6 V. In addition, the batteries for comparison are installed simultaneously to reduce interference.

**Characterization.** The infrared spectra of the samples were collected by a Thermofisher Nicolet IS50 model Fourier transform infrared spectrometer (FT-IR) with the wave number range of 4000~400 cm^-1^ and the resolution of 4 cm^-1^. Dynamic contact angle tested by Dataphysics OCA20. SEM and EDS images were taken using the Zeiss Auriga SEM/FIB system. SEM images were accelerated at 5 kV and 20 kV, and EDS measurements were accelerated at 20 kV. The atomic force microscope (AFM) was used to photograph the surface flatness test of zinc foils. The Rigaku Miniflex 600 was used to measure the XRD images of zinc electrodes. The in-situ observation of dendrites uses the Scienscope Optical Microscope, and in-situ pool of Beijing Scistar Technology Co., Ltd.

**Calculation of ionic conductivity.** The ionic conductivity (σ) was measured by a Bio-Logic VSP five-channel electrochemical workstation. The separator was soaked in electrolyte solution with two stainless steel electrode clips to test the resistance ranging from 0.01 Hz to 200 kHz, and the ionic conductivity (σ) was calculated by the following equations:^[1]^

$$\sigma=\frac{L}{R\times A}$$

where R (Ω), A (cm^2^) and L(cm) represent the resistance, area and thickness of the separator, respectively.

**Calculation of zinc anode corrosion rate.** In order to quantitatively analyze the corrosion rate of metal zinc in batteries of different systems. Firstly, a certain amount of Zn (1 mAh) is deposited on Ti foil to form Zn@Ti electrode. Zn@Ti potential shows the potential of zinc metal. Once all metal zinc is corroded by the electrolyte, the Zn@Ti electrode potential will increase significantly. The corrosion rate of zinc electrode was quantitatively analyzed by the following equation:^[2]^

$$v_{c}=\frac{m_{Zn}}{t}$$

where $m_{Zn}$ represents the mass of Zn deposited and $t$ is the corrosion time.

**Calculation of activation energy.** Activation energy E_a_ can be attained by the Arrhenius Equation. In order to study the activation energy during Zn^2+^ deposition, the electrochemical impedance spectral curves at different temperatures were calculated by Arrhenius Equation:^[3]^

$$ln\frac{1}{R_{ct}}=-\frac{E_{a}}{RT}+lnA$$

$$E_{a}=-kR=-8.314k$$

where R_ct_ is the charge-transfer resistance, A is the preexponential factor, R and T present gas constant and absolute temperature, respectively.

**Calculation of ion transfer number.** The DC polarization and AC impedance methods were applied to evaluate the zinc ion transfer number ($t_{{Zn}^{2+}}$ ) of the Zn||Zn symmetric cell, while the voltage of 20 mV was applied until the current of the battery reached a stable value. The specific value was obtained from the Bruce-Vincent-Evans equation as follows:^[4]^

$$t_{{Zn}^{2+}}=\frac{I_{S}(\Delta V-I_{0}R_{0})}{I_{0}(\Delta V-I_{S}R_{S})}$$

where ΔV (mV) is the applied polarization potential amplitude, I_0_ (mA) and I_s_ (mA) were the initial and steady-state current, respectively, and R_0_ (Ω) and R_s_ (Ω) were the initial and steady-state interfacial resistance, respectively.

**Calculation of exchange current density (*i_0_*)**. To accurately evaluate the kinetics of Zn^2+^ deposition, the exchange current density in connection with the Zn electrodeposition process can be calculated by equation:^[5]^

$$i=i_{0}\frac{zF}{RT}\eta$$

where i is the current density and η is the total overpotential; i and η can be obtained from the rate performance of symmetric cells. i0 is the exchange current density, R and F are the gas and Faradic constants, respectively, T is the thermodynamic temperature, and z is the electron number involved in the electrode reaction, where z= 2 in the Zn-ion battery.

**Calculation of Coulombic efficiency (CE).** The Coulombic efficiency (CE) was defined as below:^[6]^

$$CE=\frac{n\times Q_{C}+Q_{S}}{n\times Q_{C}+Q_{T}}\times100\%$$

where Q_T_ refers to the capacity of first Zn plating and Q_C_ refers to the capacity of Zn stripping and plating in the following n cycles from and to the initially plate Zn. The capacity observed in the last stripping sweep is named Q_S_.

**Density functional theory (DFT) calculations.** Based on the plane–wave basis sets, the Vienna ab initio simulation package (VASP) used the projector augmented–wave method^[1-2]^ to calculate the spin-polarized DFT calculations. The exchange-correlation potential was treated by using a generalized gradient approximation (GGA) with the Perdew-Burke-Ernzerhof (PBE) parametrization. The Zn (002), Zn (100), and Zn (101) crystal planes were constructed by cleaving the surface. To eliminate the influence between the periodic slab models, a 10 Å vacuum layer was established between the periodic slab models. The bottom atoms were fixed during the geometry optimization. The convergence criteria for energy, maximum force, maximum stress, and maximum displacement were 2.0×10^−5^ eV atom^−1^, 0.05 eV Å−1, 0.1 GPa, and 0.002 Å, respectively. Taking the Zn (101) crystal plane as an example, the interfacial energy ($E_{inter}$) between the HfO_2_ molecule and the Zn (101) plane was calculated using the following equation:^[7]^

$$E_{inter}=E[SZn(101)/{HfO}_{2}]-E[SZn(101)]-E\left( {HfO}_{2} \right)$$

where E[SZn(101)/ HfO_2_], E[SZn(101)] and E[HfO_2_] are the total energies of Zn (101)/ HfO_2_, Zn(101) and HfO_2_, respectively. The interfacial energies between the HfO_2_@GF separator and Zn (100) and Zn (002) planes were calculated using a consistent equation, with necessary modifications.

The adsorption energy (E_ads_) of Zn^2+^ at different positions can be calculated as:^[8]^

$$E_{ads}=E_{{Zn}^{2+}-sub}-E_{{Zn}^{2+}}-E_{sub}$$

where $E_{{Zn}^{2+}-sub}$ stands for the energy of the surface with the adsorbed molecule, $E_{{Zn}^{2+}}$is the energy of the surface, and $E_{sub}$ is the energy of the isolated molecule.

The DFT calculations are performed using the Vienna Ab initio Simulation Package (VASP), with the generalized gradient approximation (GGA) Perdew–Burke–Ernzerhof (PBE) functional to describe electron exchange and correlation. The projector-augmented plane wave (PAW) potentials are used to describe the core-valence electron interaction and take valence electrons into account using a plane wave basis set with a kinetic energy cutoff of 500 eV. The electronic energy is considered self-consistent when the energy change is smaller than 10^-5^ eV. A geometry optimization is considered convergent when the force change is smaller than 0.02 eV/Å. The diffusion of the Zn atom on the Zn (101) and in the HfO_2_ is studied by using the CI-NEB method.

**Simulation calculation of electric field distribution and concentration distribution.** COMSOL Multiphysics 6.2 is used for finite element analysis, employing the steady-state "current" physics interface and the "three-phase current distribution" physics interface to simulate the electric field, ion concentration field, and current density field. Before performing the simulation, the electrodes are preliminarily designed, including the electrode dimensions, the protrusion structure of the Zn electrode, and the structure and size of the separator. The model’s geometry includes a rectangular simulation domain of 8 µm × 6 µm, as shown in **Figure S25**. The thickness of the GF separator and the HfO_2_@GF separator is 2 µm and 3.2 µm, respectively. Three semicircular protrusions (with a diameter of 1 µm) are added to the model to realistically simulate the protrusions on the surface of the zinc anode.

In addition, we solve the Poisson equation using COMSOL Multiphysics 6.2 to simulate the impact of the separator's dielectric constant on the electric field and concentration field. The governing equation is as follows:^[9]^

$$\nabla\cdot\left( \varepsilon_{0}\varepsilon_{r}\boldsymbol{E} \right)=0$$

$$\boldsymbol{E}=-\nabla\varphi$$

where $\varepsilon_{0}$ is the vacuum dielectric constant, $\varepsilon_{r}$ is the relative dielectric constant, $\boldsymbol{E}$ is the electric field, and $\varphi$ is the potential. The dielectric constants of GF separators and HfO_2_@GF separators are measured by the broadband dielectric spectrometer and the values of dielectric constants at 40 Hz (2.58 and 7.17) are selected for simulation, respectively.

The ion concentration followed Fick’s First Law for diffusion, the electromigration followed the Nernst-Planck relationship, and the reaction on the electrode surface followed the Butler-Volmer equation.^[10]^ The boundary conditions are the potentials of the positive and negative electrodes, with the positive electrode set as the zero-potential boundary and the negative electrode potential set to the polarization voltage of the symmetric battery determined experimentally. The initial Zn^2+^ concentration is set to 2 M. The current density through the battery is set to 2 mA cm^-2^. The system temperature is set to 298 K. The model is solved using the Newton-Raphson method in the COMSOL Multiphysics solver, with a relative error of less than 1×10^-9^.

**Supplementary Figures**

**Figure S1.** Long cycling performance of HfO_2_@GF separators with different doping ratios at 5 mA cm^-2^ and 1 mAh cm^-2^.

**Figure S2.** Optical photographs and SEM images of (a) GF and (b) HfO_2_@GF separators.

**Figure S3.** (a) Low and (b) high magnification SEM images of the pure PVDF@GF separator. (c) Low and (d) high magnification SEM images of the pure HfO_2_@GF separator.

**Figure S4.** Long-cycle performance comparison of Zn||Zn symmetric batteries with different separators at 5 mA cm^-2^, 1 mAh cm^-2^.

**Figure S5.** Optical photographs and SEM images of (a) GF and (b) HfO_2_@GF separators.

**Figure S6.** Static contact angle of 2 M ZnSO_4_ solution on pure PVDF@GF separator and pure HfO_2_@GF separator.

**Figure S7.** Contact angle comparison of HfO_2_@GF separator with those reported so far for aqueous zinc-based batteries.

**Figure S8.** Fitted Raman spectra of the –OH bond in the infiltrated solution of the right chamber with different separators. (The left chamber of the electrolysis cell consists of 10 mL of 2 M ZnSO_4_ solution. After standing for 3 h, the infiltrated solution from the right chamber is collected.)

**Figure S9.** Schematic diagram of an ion permeation device (left: 10 mL 2 M ZnSO_4_ solution; right: 10 mL deionized water).

**Figure S10.** (a) Schematic of constant-current charging of the electrolyzer ( left: 2 M ZnSO_4_ solution; right: deionized water). (b) Right chamber ion concentration measured by ICP-OES.

**Figure S11.** The tensile stress-strain curves of GF separator and HfO_2_@GF separator.

**Figure S12.** Optical images and surface SEM images of the (a) GF separator and (b) HfO_2_@GF separator after 50 cycles.

**Figure S13.** (a) Voltage-time curves of Zn||Zn@Ti cells in different separators. Zn@Ti electrodes are formed by depositing a certain amount of metallic zinc (1 mAh) on the Ti foil. (b) Zn anodic corrosion rates with different separators.

**Figure S14.** CV curves of the Zn||Ti asymmetric cells with GF separator and HfO_2_@GF separator at 1 mV s^-1^.

**Figure S15.** Nucleation overpotential curves of Zn||Cu cells with different separators at different current densities.

**Figure S16.** EIS curves of Zn||Zn symmetric batteries with (a) GF separator and (b) HfO_2_@GF separator varying with temperature.

**Figure S17.** The number of Zn^2+^ ion transfer ($t_{\mathrm{Zn}^{2+}}$) of Zn||Zn symmetric batteries with (a) GF separator and (b) HfO_2_@GF separator.

**Figure S18.** The computation models for interfacial energy calculations of the HfO_2_@GF separator with Zn (101), Zn (002), and Zn (100) crystal planes, respectively.

**Figure S19.** The side and top views of the computational model for the adsorption of Zn^2+^ on the Zn (101) crystal surface.

**Figure S20.** The side and top views of the computational model for the adsorption of Zn^2+^ at different sites on the HfO_2_ crystal.

**Figure S21.** The surface SEM morphology of the zinc anodes with (a) GF separator and (b) HfO_2_@GF separator after 50 cycles at a current density of 5 mA cm^-2^.

**Figure S22.** Surface SEM images and EDS energy spectra of zinc anode with GF separator after 50 cycles at 5 mA cm^-2^ and 1 mAh cm^-2^.

**Figure S23.** Surface SEM images and EDS energy spectra of zinc anode with HfO_2_@GF separator after 50 cycles at 5 mA cm^-2^ and 1 mAh cm^-2^.

**Figure S24.** Schematic diagram of zinc crystal structure.

**Figure S25.** GIXRD patterns of zinc anodes after 50 cycles in different separators.

**Figure S26.** Simplified geometric models of (a) GF separator and (b) HfO_2_@GF separator in COMSOL simulation.

**Figure S27.** Simulation of interfacial current density distribution of zinc anode with GF and HfO_2_@GF separator.

**Figure S28.** The long cycle performance of Zn||Zn symmetric cells at (a) 1 mA cm^-2^, 1 mAh cm^-2^ and (b) 2 mA cm^-2^, 1 mAh cm^-2^.

**Figure S29.** The long cycle performance of Zn||Zn symmetric cells with (a) ZnO@GF separator and (b) SiO_2_@GF separator at 5 mA cm^-2^ and 1 mAh cm^-2^.

**Figure S30.** The SEM image of the cathode active material ZVO.

**Figure S31.** The long-cycle performance of Zn||ZVO batteries with different separators at 10C.

**Figure S32.** The rate performance of Zn||ZVO batteries with different separators.

**Figure S33.** The CV curves of Zn||I_2_ batteries with different separators at a scan rate of 0.1 mV s^-1^ for different cycles.

**Figure S34.** The CV curves of Zn||I_2_ batteries with GF separator at different scan rates.

**Figure S35.** The peak fitting of the CV curves for Zn||I₂ batteries with a) GF separator and b) HfO_2_@GF separator at different scan rates.

**Figure S36.** Capacity-voltage curves of battery with GF separator at different rates.

**Figure S37.** The self-discharge test of Zn||I_2_ batteries with GF separator and HfO_2_@GF separator.

**Supplementary Tables**

**Table S1.** The ionic conductivity parameters of GF separator and HfO_2_@GF separator in 2 M ZnSO_4_ electrolyte.

| **Separator** | **R (Ω)** | **A (cm^2)^** | **L(μm)** | **ionic conductivity (σ)** |
| --- | --- | --- | --- | --- |
| GF | 1.89 | 1.13 | 250 | 11.71 mS cm^-1^ |
| HfO_2_@GF | 1.60 | 1.13 | 270 | 13.83 mS cm^-1^ |

**Table S2.** R_ct_ of Zn||Zn symmetric cells with GF separator and HfO_2_@GF separator at different temperatures.

| **Separator** | **10℃** | **20℃** | **30℃** | **40℃** | **50℃** | **60℃** | **E_a_**  **(kJ mol^-1^)** |
| --- | --- | --- | --- | --- | --- | --- | --- |
| GF | 2086.65 | 1404.96 | 791.94 | 548.20 | 315.85 | 192.21 | 37.512 |
| HfO_2_@GF | 1196.77 | 840.18 | 524.27 | 356.46 | 226.99 | 147.86 | 33.029 |

**Table S3.** Calculation of transference numbers from analysis of polarization experiments.

| **Separator** | **∆V(mV)** | **R_0_(Ω)** | **R_s_(Ω)** | **I_0_(mA)** | **I_s_(mA)** | $\mathbf{t}_{\mathbf{Zn}^{\mathbf{2+}}}$ |
| --- | --- | --- | --- | --- | --- | --- |
| GF | 20 | 122 | 340 | 0.1963 | 0.0741 | 0.287 |
| HfO_2_@GF | 20 | 155 | 315 | 0.2587 | 0.0852 | 0.729 |

**Table S4.** Comparison of current density, areal capacity and cycle life of Zn||Zn symmetric cells with various modified Zn-based separators.

| **Separator** | **Current density**  **(mA cm^-2^)** | **Areal capacity**  **(mAh cm^-2^)** | **Cycle life (h)** | **Ref.** |
| --- | --- | --- | --- | --- |
| PTFE | 1 | 1 | 1300 | *Adv. Funct. Mater.* **2024**, *34*, 2316619 |
| PAN@SBMA | 1 | 1 | 1700 | *Adv Adv. Funct. Mater.* **2024**, *34*, 2408863 |
| UC/GF | 5 | 1 | 3400 | *Adv. Funct. Mater.* **2024**, *34*, 2410712 |
| FACNF | 2 | 1 | 1600 | *Small* **2024**, *20*, 2311203 |
| PVDF@GF | 10 | 1 | 500 | *Small* **2024**, *20*, 2305119 |
| CG | 2 | 1 | 1750 | *Adv. Energy Mater.* **2021**, *11*, 2101299 |
| MZnHCF-GF | 2 | 1 | 2700 | *Adv. Sci.* **2024**, *11*, 2407410 |
| ANF | 2 | 1 | 1470 | *Adv. Energy Mater.* **2024**, *14*, 2401858 |
| CCM | 5 | 1 | 600 | *Adv. Energy Mater.* **2023**, *13*, 2302126 |
| Janus | 10 | 1 | 600 | *Adv. Mater.* **2020**, *32*, 2003425 |
| PT-GF-PT | 5 | 1 | 2200 | *Small* **2024**, *20*, 2307357 |
| **HfO_2_@GF** | **1** | **1** | **2035** | **This work** |
| **HfO_2_@GF** | **2** | **1** | **3513** | **This work** |
| **HfO_2_@GF** | **5** | **1** | **4660** | **This work** |
| **HfO_2_@GF** | **10** | **1** | **5000+** | **This work** |

**Table S5.** Overpotentials of the GF and HfO_2_@GF separator symmetrical cells under different current densities and linear fits between current density and overpotential.

| **Current density *i***  **(mA cm^-2^)** | **Overpotential η (mV)** | | |
| --- | --- | --- | --- |
|  | **GF separator** | **HfO_2_@GF separator** | |
| 1 | 40.7 | | 30.2 |
| 2 | 51.6 | | 35.9 |
| 4 | 77.6 | | 46.8 |
| 6 | 86.7 | | 59.5 |
| 8 | 110.6 | | 70.6 |
| 10 | 130.1 | | 80.6 |
| Linear fit | $\eta_{1}=9.71i+32.70$ | | $\eta_{2}=5.67i+24.63$ |

**References**

[1] D. Zhang, H. Lu, C. Duan, Y. Qin, Z. Zhu, Z. Zhang, N. Lyu, Y. Jin, *Small* **2024**, *20*, 2307357.

[2] Y. Wang, Z. Wang, W. K. Pang, W. Lie, J. A. Yuwono, G. Liang, S. Liu, A. M. D. Angelo, J. Deng, Y. Fan, K. Davey, B. Li, Z. Guo, *Nat. Commun.* **2023**, *14*, 2720.

[3] Z. Zhu, H. Lu, D. Zhang, X. Jiang, C. Duan, Y. Qin, X. Yuan, Y. Jin, *J. Energy Storage* **2024**, *92*, 112211.

[4] C. Duan, H. Lu, D. Zhang, Z. Zhu, Y. Qin, X. Yuan, Y. Jin, *Chem. Eng. J.* **2024**, *487*, 150413.

[5] H. Lu, D. Zhang, Q. Jin, Z. Zhang, N. Lyu, Z. Zhu, C. Duan, Y. Qin, Y. Jin, *Adv. Mater.* **2023**, *35*, 2300620.

[6] Z. Liu, R. Wang, Y. Gao, S. Zhang, J. Wan, J. Mao, L. Zhang, H. Li, J. Hao, G. Li, L. Zhang, C. Zhang, *Adv. Funct. Mater.* **2023**, *33*, 2308463.

[7] L. Yang, Y.-J. Zhu, H.-P. Yu, Z.-Y. Wang, L. Cheng, D.-D. Li, J. Tao, G. He, H. Li, *Adv. Energy Mater.* **2024**, *14*, 2401858.

[8] Y. Tan, D. Chen, T. Yao, Y. Zhang, C. Miao, H. Yang, Y. Wang, L. Li, V. Kotsiubynskyi, W. Han, L. Shen, *Adv. Sci.* **2024**, *11*, 2407410.

[9] S. Zhou, X. Meng, C. Fu, D. Xu, J. Li, Q. He, S. Lin, S. Liang, Z. Chang, A. Pan, *Small* **2023**, *19*, 2303457.

[10] M. Liu, W. Yuan, X. Qu, X. Ru, X. Li, T. Wang, X. Wang, Y. Wang, Y. Liu, N. Zhang, *Energy Environ. Sci.* **2024**, *17*, 9611.
